# Mapping the SARS-CoV-2–Host Protein–Protein Interactome by Affinity Purification Mass Spectrometry and Proximity-Dependent Biotin Labeling: A Rational and Straightforward Route to Discover Host-Directed Anti-SARS-CoV-2 Therapeutics

**DOI:** 10.3390/ijms22020532

**Published:** 2021-01-07

**Authors:** Rosa Terracciano, Mariaimmacolata Preianò, Annalisa Fregola, Corrado Pelaia, Tiziana Montalcini, Rocco Savino

**Affiliations:** 1 Department of Experimental and Clinical Medicine, University “Magna Græcia”, 88100 Catanzaro, Italy; tmontalcini@unicz.it; 2 Department of Health Sciences, University “Magna Græcia”, 88100 Catanzaro, Italy; preiano@unicz.it (M.P.); annalisafregola@gmail.com (A.F.); 3 Respiratory Medicine Unit, University “Magna Græcia”, 88100 Catanzaro, Italy; pelaia.corrado@gmail.com; 4 Department of Medical and Surgical Sciences, University “Magna Græcia”, 88100 Catanzaro, Italy

**Keywords:** SARS-CoV-2, COVID-19, affinity-purification mass spectrometry, proximity-dependent biotin labeling (BioID), protein–protein interaction, proteomics, drug repurposing, virus, antiviral, emerging

## Abstract

Protein–protein interactions (PPIs) are the vital engine of cellular machinery. After virus entry in host cells the global organization of the viral life cycle is strongly regulated by the formation of virus-host protein interactions. With the advent of high-throughput -omics platforms, the mirage to obtain a “high resolution” view of virus–host interactions has come true. In fact, the rapidly expanding approaches of mass spectrometry (MS)-based proteomics in the study of PPIs provide efficient tools to identify a significant number of potential drug targets. Generation of PPIs maps by affinity purification-MS and by the more recent proximity labeling-MS may help to uncover cellular processes hijacked and/or altered by severe acute respiratory syndrome coronavirus 2 (SARS-CoV-2), providing promising therapeutic targets. The possibility to further validate putative key targets from high-confidence interactions between viral bait and host protein through follow-up MS-based multi-omics experiments offers an unprecedented opportunity in the drug discovery pipeline. In particular, drug repurposing, making use of already existing approved drugs directly targeting these identified and validated host interactors, might shorten the time and reduce the costs in comparison to the traditional drug discovery process. This route might be promising for finding effective antiviral therapeutic options providing a turning point in the fight against the coronavirus disease-2019 (COVID-19) outbreak.

## 1. Introduction

The global health emergency for the current worldwide outbreak, caused by the novel severe acute respiratory syndrome coronavirus 2 (SARS-CoV-2) virus since December 2019, has posed the urgent requirement to discover specific treatments to stop the pandemic. Fortunately, international efforts from scientific community have provided an extraordinary wealth of studies which have greatly contributed to better elucidate the mechanism of pathogenicity of SARS-CoV-2 [1]. In particular, it has been demonstrated that SARS-CoV-2 uses the receptor angiotensin-converting enzyme 2 (ACE2) for entry and the transmembrane serine protease 2 (TMPRSS2) for spike glycoprotein (S protein) priming [2]. Further structural studies have disclosed the mechanism for the recognition of SARS-CoV-2 by full-length ACE2 [3].

Providing new insights into viral transmission might help to reveal important therapeutic targets and to strongly contribute to the emerging and rapidly evolving field of coronavirus disease 2019 (COVID-19) drug discovery [4,5]. It is well known that viral infections mostly depend on viruses’ ability to specifically interact with host proteins through a complex network of interactions. To play their physiological and defense role, proteins expressed in eukaryotic cells establish a regulated network of protein–protein interactions (PPIs). From a systems biology point of view, the interplay between the PPI network of the virus (intraviral interactome) and the PPI network of the host (host interactome) generates a new system, the virus–host interactome, of primary importance in defining the strategy of the virus to complete its own lifecycle, evading the immune host defense and eventually causing several diseases in the host [6,7]. Moreover, one of the most relevant features of viral evolution is the high mutation rate, which frequently leads to virus adaptation to a new host [8]. Although vaccination has already started in UK and other countries, this may not be a definitive solution to the pandemic because there is always the possibility that the above-mentioned high mutation rate might lead to the generation of new virus strains escaping vaccine-induced immune protection. Therefore, identification and characterization as well as systematic analysis of virus–host interactome can be of tremendous value not only to uncover virus pathogenic strategies but also to search novel antiviral therapeutic approaches targeting the virus–host interactome. Presently, there are several strategies to map PPIs which are based either on in vitro experiments [9] or on literature mining [10]. Among experimental approaches, while yeast-two hybrid screens determine direct binary interactions [11], genomic wide screens [12,13] and proteomics approaches such as affinity-purification mass spectrometry (AP-MS) and proximity-dependent biotin labeling (BioID) coupled to MS provide more comprehensive interaction maps [14]. The large datasets of PPIs generated by these approaches may reveal new host-virus interactions easily targetable by host-directed therapies on the basis of already existing small-molecule candidates. The so called “repurposing” or “repositioning” of pharmaceutical drugs and preclinical drugs, assisted by PPI maps, might represent a shortcut for the rapid identification of promising druggable targets to promptly manage SARS-CoV-2 infection. AP-MS and related proteomics approaches have been focused on coronaviruses in the past to define the structure-function relationships of viral proteins, the alterations of host proteomes upon viral infection and the viral–protein interactomics to decipher the wide interaction networks between viral and host proteins [15,16].

Maps of PPIs between SARS-CoV-2 proteins and human proteins have been very recently identified (determined/uncovered) by both AP-MS [17,18,19,20,21] and BioID approaches [22,23,24]. The intriguing and fascinating SARS-CoV-2 landscapes emerging from these studies offer an extraordinary springboard for the systematic exploration of the virus–host interface in the search of host proteins already targeted by existing drugs. This review provides an in-depth description and discussion on relevant results of these recently published SARS-CoV-2 PPIs proteomics datasets. A preliminary description of AP-MS and BioID technologies will be provided aiming at clarifying and simplifying this topic to a wider multidisciplinary readership. Next, we furnish an in-depth analysis of the published datasets and conclude this paper with perspectives towards future follow-up experiments and further investigations which might provide a valid support for the rapid development of host-directed anti-SARS-CoV-2 therapeutics.

### 1.1. Structural Features of SARS-CoV-2

SARS-CoV-2 is an enveloped virus containing a positive-sense single-stranded RNA. According to the recently published genome sequence, SARS-CoV-2 can be considered a member of the *Coronaviridae* family as it shows typical features of other coronaviruses, sharing ~80% sequence identity with SARS-CoV [25]. Its genome encodes four conserved structural proteins: spike (S) glycoprotein, small envelope (E) glycoprotein, membrane (M) glycoprotein, and nucleocapsid (N) protein; although hemagglutinin-esterase protein is present in a subset of beta coronaviruses, so far no such coding region has been found in the SARS-CoV-2 genome [25,26]. The genome also encodes several accessory proteins. In particular, the reported annotation of SARS-CoV-2 [26] contains one ORF1ab, encoding a polyprotein which is further processed to generate sixteen non-structural proteins (Nsp1–16), and 9 ORFs encoding accessory proteins: ORF3a, ORF3b, ORF6, ORF7a, ORF7b, ORF8, ORF9b, ORF14 (referred as ORF9c by Gordon et al. [17,18]) and ORF 10 [25].

Structural proteins are not just components of the capsid and the envelope, but they also play an important role in the viral life cycle. The S protein is a transmembrane protein positioned in the outer portion of the virus assembled in homotrimers protruding from the viral surface like the spikes of a crown. The peculiar structure of the S protein mediates the virus entry inside the host cell. In fact, it forms a complex with host cells surface receptor ACE2 promoting the fusion between virion envelope and the cell membrane, which also involves S cleavage operated by TMPRSS2 [27]. Following the entrance in the host cell, the virus liberates the nucleocapsid containing the viral genome into the cytoplasm. Next, the viral RNA is first translated to synthetize the Nsps responsible for the RNA-dependent RNA polymerization, which in turn, synthetize both new genomic RNAs and also the subgenomics mRNAs necessary for the translation of the structural proteins (S, E, M, N: assembly of new viral particles) and of the accessory proteins (ORF3a-ORF10) [28]. In particular, the Nsp factors form the replicase–transcriptase machinery, which include the enzymatic activity essential for viral RNA replication (Nsp12, Nsp7, Nsp8, Nsp13, Nsp14) and viral protein translation (Nsp14 and Nsp16). Nsps may act also as virulence factors inhibiting the host immune system defenses (Nsp1, Nsp15) [28].

Many other information on novel functions of these proteins might be revealed by studies on both intraviral PPI and viral-host protein as will be discussed in Section 2 of this review.

### 1.2. AP-MS and BioID

The rapidly expanding approaches of MS-based proteomics in the study of PPI provides an efficient tool to discover a significant number of potential drug targets. In this section, we furnish a brief general description of AP-MS and BioID coupled to MS, the two approaches used to map SARS-CoV-2- host protein–protein interactomes.

In order to exert their functions, proteins establish interactions with other proteins, forming complexes and high-order network structures that control cellular physiology. Therefore, the identification of the binding interactors in a protein complex is of crucial importance in defining the molecular machinery of cells at the protein level and in understanding specific cellular physiological pathway. At the same manner, the interactions established between viral proteins and host proteins, after virus entrance in a specific host cell, are fundamental for the virus to progress through its lifecycle. Therefore, studies disclosing interactions between viral and host proteins may provide useful evidence on the mechanism that the viruses employ to hijack cellular machinery for their benefit [29,30,31,32]. Currently, AP-MS is a widely used tool for the characterization of PPIs [9,33,34] and for studying mechanisms of infected cell rewiring [35,36].

**Figure 1:**
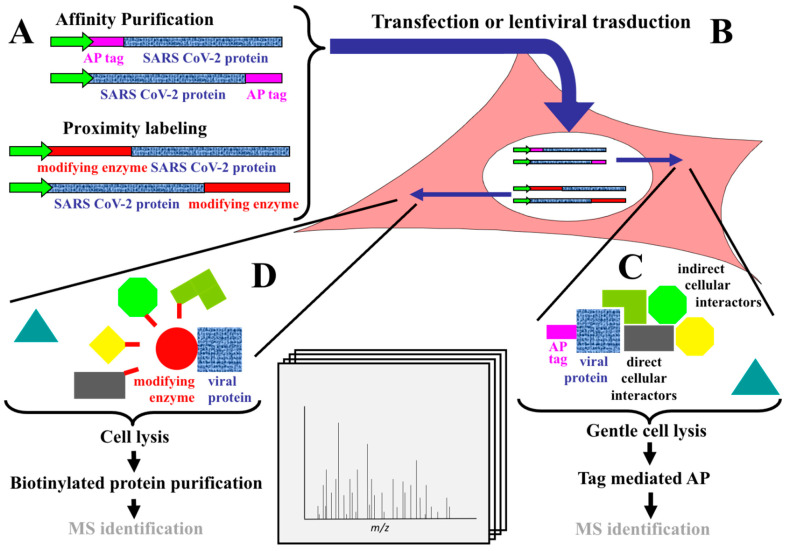
Schematic representation of (protein–protein interaction) PPI identification strategies. (**A**) In the Affinity Purification (AP) strategy, the sequence coding for the viral protein (shown in blu) is fused in frame, either at the N- or at the C-terminus, with a peptide tag (shown in purple) which, for the investigations here reviewed, can be Strep [17,18], FLAG [19,20] or HA [21]. In the proximity labeling strategy, the sequence coding for the viral protein is fused in frame, either at the N- or at the C-terminus, with a biotin protein ligase modifying enzyme (shown in red). In all cases, a promoter (green arrow) drives the expression of the fusion protein. (**B**) The constructs described in (**A**) are transferred to the test cell line which, for the investigations here reviewed, can be HEK-293 cells [17,18,19,20,23,24] or A549 cells [21,22] by mean of transfection (either transient [17,18,19,20] or stable [23,24]) or by lentiviral mediated transduction [21,22]. In the test cell line, the promoter of the construct drives the expression of the fusion protein. (**C**) In the AP strategy, following gentle cell lysis, the cellular preys (here shown in different colors and shapes) interacting, either directly or indirectly, with the viral bait are affinity purified, usually using a monoclonal antibody specific for the AP tag (shown in purple). If a cellular protein (indicated by a triangle) does not interact with the viral bait, it is not affinity purified at this stage. Finally, after AP, specifically bound proteins are digested and identified by mass spectrometry (MS). (**D**) In the proximity labeling approach, after expression of the fusion protein (here shown as a blue square, representing the viral protein, fused with a red circle, representing the modifying enzyme) in the test cell, the biotin protein ligase modifying enzyme mediates the “promiscuous” biotinylation (shown in red) of proteins (here shown in different colors and shapes) in close proximity (~5–10 nm) to the enzyme. If a cellular protein (indicated by a triangle) is not in proximity with the enzyme-viral bait fusion protein, it is not biotinylated. Following cell lysis, only biotinylated proteins are purified by streptavidin and then identified by MS.

In general, AP is based on the selective binding of a genetically fused affinity tag. In the studies here reviewed, the viral proteins are “affinity tagged” in order to identify their binding partners present in the specific host cellular system. To specifically construct SARS-CoV-2 PPI maps in several reports AP-MS was used [17,18,19,20,21], while in other studies the authors [22,23,24] used the different technology BioID in combination with MS [37]. The main steps of workflows describing these two approaches are illustrated in Figure 1.

In AP-MS, according to the widely used protocols, the cells (HEK293 [17,18,19,20], A549 [21]), are transfected with a plasmid [17,18,19,20] or transduced with a lentiviral construct [21] coding for a specific bait virus protein fused to a tag (Figure 1).

Affinity tags are generally peptides or proteins and can be small or large tags depending on their size. FLAG and haemagglutinin (HA), used in the studies here reviewed, are formed by a short peptide chain and, in comparison to larger tags, offer the advantage, to have a less impact on protein size, and consequently affect with a less extent both its interactions and localization. After a reasonable expression time, cells are lysed and the tagged bait, together with bounded associated partners (preys), is isolated using a tag-specific antibody or a specific ligand (either biological or chemical) bound to a solid support [33]. After AP, specifically bound proteins (i.e., proteins not recovered in the control experiment) are digested and identified by liquid chromatography (LC)-MS/MS (Figure 1). However, one limitation of this approach may be due to the formation during cell lysis (or in non-native conditions) of nonspecific interactions, which may sometimes include in the complex non-physiological targets. Additionally, this tool is not properly suitable for the detection of protein complexes in which a weak affinity interaction is established between the interactors and/or for complexes characterized by fast kinetic of dissociation, for example transient interactions occurring in post translational modifications (PTMs) or those occurring at poorly soluble membranes proximity. In these cases, the specific interaction might be lost if stringent rinsing procedures are used during affinity chromatography [9,33]. An alternative option to overcome these hurdles was proposed introducing the proximity-dependent biotinylation assay described for the first time by Roux et al. [38]. It is well known that the addition of biotin to protein is a highly specific enzyme-catalyzed process [39]. However, Choi-Rhee and colleagues demonstrated that also nonspecific biotinylation of proteins can occur [40]. The proximity-dependent biotinylation is a smart tool (BioID) based on nonspecific biotinylation; in particular, BioID uses the activity of BirA* (a R118G mutant of the Escherichia coli biotin protein ligase) to mediate the “promiscuous” biotinylation of proteins. The reaction has been suggested to involve the release and the diffusion in solution of biotinoyl-5′-AMP (a reactive activated biotin intermediate), with the modification of exposed lysine on the target proteins in a nonspecific modality. In other words, this diffusion generates a “cloud” of reactive biotin molecules which, in turn, can react with lysines residues of adjacent proteins, with the consequent biotinylation of neighboring proteins in the physiological cellular environment [39,40]. Only the biotinylated proteins are then purified by streptavidin (Figure 1) AP and then analyzed by MS. In this case, the use of harsh denaturing conditions, because of the high affinity of the streptavidin–biotin bond, do not compromise the biotin tag because covalently bound to the host proximity partner. Additionally, the use of stringent conditions effectively solubilizes most cellular proteins and also destroys nonspecific binding and post-lysis formation of non-physiologic protein complexes. Newer versions of this assay, called TurboID [41,42] and mini-TurboID [43] can catalyze the proximity-dependent biotinylation on a timescale of minutes instead of several hours. Specifically, both BirA* and the mini-Turbo version of this tool have been used to study the proximity interaction landscape of SARS-CoV-2 in three studies discussed in the following section [22,23,24]. However, although BioID strategy is well suited for identifying transient interactors, this added value is counterbalanced by decreased specificity.

### 1.3. Data Filtering and Graphical Network Representation

Investigational studies of PPIs, as usually carried out in AP-MS experiments, during the lysis step (Figure 1) may bring together proteins from different cellular compartments in situations that do not really reflect the real in vivo environmental conditions. Consequently, this can lead to the detection of artefactual interactions or false positives. The use during protein interaction analysis of stringent filtering in order to select a rank for the confidence of interactions (for example “potential” and/or “high-confidence” interactions) and of other precautions such as the exclusion of baits showing unusually high number of background proteins (see [17]) is necessary in these studies for the reasons reported above [44]. Given the inherent limitation of these methodologies, the large MS datasets of interactions obtained are rigorously filtered to obtain a “core” dataset of PPIs. To accurately evaluate consistency of PPI datasets obtained by AP-MS or by proximity labeling several groups use online resources of integrated databases of biological interaction data for most major model organisms and software computational/scoring methods [45]. For example, identified proteins from Krogan group [17,18] were subjected to a PPI score filtering with both SAINT express (v.3.6.3) [46] and MiST (https://github.com/kroganlab/mist) software [31,47]. The MiST (Mass spectrometry interaction STatistics) is a computational algorithm used to process large scale MS raw data following AP, by performing quality controls in replicated AP-MS experiments and assigning a rank of interactions to biologically pertinent bait-prey pairs [48]. MiST was also used in several studies here described [17,18,20] to calculate the statistical significance of the dataset.

Generally the proteomics datasets generated by the methods herein described, are then converted in network of interactions graphically visualized by Cytoscape, an open source software platform that provides an explicative picture of the networks of interactions and pathways with annotations, gene expression profiles and other state data (https://cytoscape.org/what_is_cytoscape.html) [49].

Another important resource that also supports these PPI studies is Gene Ontology (GO) (http://geneontology.org/) [50]. In fact, GO allows the computational representation of the global information on gene functions from humans and from many other different organisms. GO is mainly used for an in depth interpretation of large proteomics or other -omics datasets. Many of the research groups here reviewed have also used this “Gene Ontology enrichment analysis” for applying statistical tests and finding whose expression alteration might suggest a correlation with disease phenotypes **(**http://geneontology.org/docs/go-enrichment-analysis/).

### 1.4. Validation Experiments

Once identified and filtered high-confidence interactions, (particularly those of main interest), need to be corroborated by orthogonal experimental methods. A conventional method is provided by classical immunoprecipitation (IP) experiments. An alternative approach for validating an interaction is the assessment of the co-localization of the proteins by immunofluorescence (IF) microscopy. This approach informs whether the proteins have the opportunity to interact by co-existing in the same subcellular compartment, which is important for all interaction studies. IF microscopy can assess the co-localization of proteins within several hundred nanometers. Noteworthy, the IP experiments and the co-localization by IF lack the ability to distinguish between indirect and direct interactions.

### 1.5. Mapping PTMs Profiles

In order to more comprehensively uncover the SARS-CoV 2-host PPI landscape, concomitant and collateral multilevel -omics studies associated to main stream AP-MS investigations are a very useful complement. In particular, a wealth of information can be obtained by assessing changes observable in the host proteome, transcriptome, ubiquitinome and phosphoproteome in response to virus infection. In fact, as already underlined, several viruses remodel host cellular pathways so as to replicate and evade host cell responses. Thus, targeting cellular PTMs pathways offers a mechanism by which viruses can use to promptly change a hostile cellular environment into a hospitable one. In particular, many PPIs between virus ad host proteins are driven by phosphorylation events, therefore the contribution of phosphoproteomics is essential to identify druggable targets such as kinases and kinase substrates. Briefly, as a general workflow, the host cells are infected with SARS-CoV-2 and are then harvested at time 0 (prior to the infection) and at different time points after viral uptake. After cell lysis, proteins are digested into peptides and changes of protein expression upon infection are assessed by label-free quantitative proteomics for the studies on which this review is focused. To generate phosphoproteome data, samples of digested peptides are enriched for phosphorylated peptides before MS analysis and, using data-dependent acquisition and/or data-independent acquisition approach, each sample is analyzed for changes in phosphorylation. All identified phosphorylation sites and protein identifiers are then mapped to their respective corresponding human protein orthologs, in the case of infection experiments performed in non-human cells. The label-free quantification approach used in these phosphoproteomics surveys enables, as outlined in the following sections, the accurate identification with a high-quality quantification of phosphorylation sites. Moreover, as will be also discussed in the following sections, phosphoproteomic studies can lead to the selection of small molecules targeting dysregulated pathways eliciting effective antiviral efficacy.

## 2. Mapping the SARS-CoV-2 Interactome

In the following sections we systematically report on SARS-CoV-2 PPI maps uncovered through AP-MS [17,18,19,20,21] or BioID [22,23,24]. Table 1 summarizes, for each study, the cell lines, the method used for generation of PPI maps and other experimental settings. Additionally, the Table 1 also resumes main results from network enrichment analysis, links to data availability and web resources.

**Figure 2:**
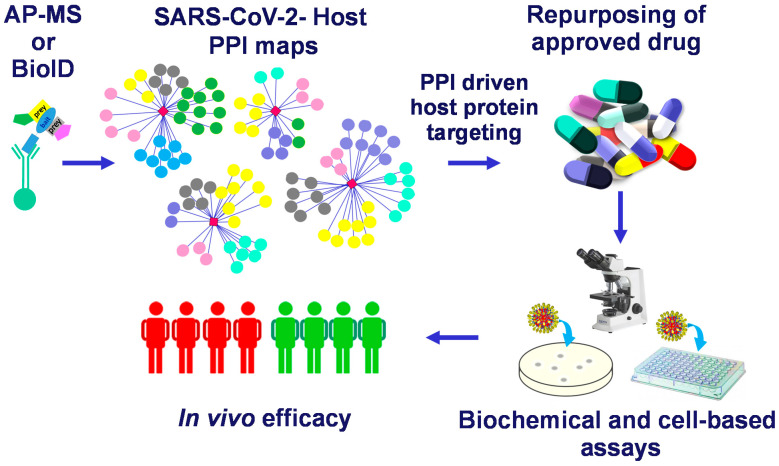
Overview of PPIs-based drug repurposing. SARS-CoV-2 interactomes are generated by affinity purification Mass Spectrometry (AP-MS) or by Proximity-Dependent Biotin Labeling (BioID). Interactions between SARS-CoV-2 viral baits proteins and host proteins targetable by already approved drugs are selected. Next, druggable host targets are analysed by biochemical and cell-based assays for antiviral activity. Selected lead compounds with in vitro anti-viral efficacy are then tested for in vivo efficacy on COVID-19 patients.

Among the SARS-CoV-2 interactomes obtained by AP-MS or by BioID approaches, specific interactions between SARS-CoV-2 viral baits proteins and host proteins are selected for repurposing approved drugs. Identified candidates are then analyzed by biochemical and cell-based assays. Selected drugs with anti-viral efficacy are finally tested in clinical assays; the advantages of drug repurposing are a reduction of the long times required for regulatory agencies approval and of the costs which are very high (billions of dollars) in a de novo drug discovery process [51]. The general workflow of drug repurposing strategy based on PPI datasets analysis just described is schematically summarized in Figure 2.

### 2.1. Mapping the SARS-CoV-2 Interactome Generated in HEK-293 Cells by AP-MS

In a pioneering proteomic/chemoinformatic study, Gordon and colleagues first uncovered the SARS-CoV-2 interactome characterized by the human host proteins physically associated with each of the SARS-CoV-2 proteins [17]. In particular, they first cloned, tagged and expressed (by transient transfection) in human embryonic kidney HEK293T/17 cells all the SARS-CoV-2 proteins (with the exception of Nsp3 and Nsp16) and then identified by MS 332 human proteins physically associated with the viral proteins using the AP-MS approach. MS datasets have been archived to the ProteomeXchange Consortium through the PRIDE repository partner (Table 1). High-confidence interacting hosts were filtered by the scoring algorithms SAINT express [46] and MiST [31]. Additionally, the authors uploaded the PPI networks to the public resource NDEx (https://public.ndexbio.org/#/network/43803262-6d69-11ea-bfdc-0ac135e8bacf).

Each viral protein was analyzed for GO enrichment analysis revealing the main cellular processes of the interacting proteins; therefore, this study provided the interaction maps of all the studied SARS-CoV-2 proteins, suggesting their involvement in several cellular pathways, including protein trafficking, ubiquitination regulation, transcription and, in particular, translation (Table 1). Among others, the authors identified a high-confidence interaction between the epigenetic regulator histone deacetylase 2 (HDAC2) and the wild-type Nsp5 main protease and, importantly, a cleavage site for the Nsp5 viral protease was predicted between the nuclear localization sequence (NLS) of HDAC2 and the HDAC2 domain; the authors speculated that, separating the HDAC2 domain from the HDAC2 NLS by proteolytic cleavage, the viral Nsp5 protease might inhibit HDAC2 transport into the nucleus and consequently prevent HDAC2 mediating both inflammation and interferon response. Interestingly, HDAC2 is targeted by the approved drug valproic acid, which was tested by the authors in two different in vitro systems [17]. Although valproic acid resulted inactive in the in vitro experiments, it might still demonstrate efficacy in more complex systems as in the case of indomethacin, studied by the same group in a different investigation summarized below [18]. Other important interactions observed in this study were also those of SARS-CoV-2 viral proteins Nsp13 with TBK1 and TBKBP1, Nsp15 with RNF41/Nrdp1 and Orf9b with *TOMM70*, which are proteins of the interferon (IFN) immune pathway. Other innate immune signaling proteins TLE1, 3, and 5 and NLRX1, F2RL1, NDFIP2, all related to NF-κB pathway, were targeted by Nsp13 and Orf9c, respectively. Interestingly, the authors also detected the interaction between the viral protein Orf6 and NUP98-RAE1, an interferon-inducible mRNA nuclear export complex, that it is hijacked or degraded by several viruses, as demonstrated in previous investigations [52,53,54,55]. Additionally, previous X-ray diffraction studies, have shown structural features in the complex between the M protein of vesicular stomatitis virus and NUP98-RAE1 [56], that were found conserved in the C-terminal region of Orf6 protein in SARS-CoV-2, adding credit to the observed interaction. Moreover, since SARS-CoV protein Orf6 antagonizes host interferon signaling, by sequestering nuclear import factors [57], the authors suggest that with a similar mechanism NUP98-RAE1 interaction with Orf6 can accomplish the same function in the case of SARS-CoV-2. Indeed, the same interaction has been detected also in several other investigations here reviewed, as summarized in Section 3 below.

Interestingly, although the interactors were identified in kidney originating cells HEK-293, which are not the physiological target of infections, the results remain highly relevant as the same proteins were found to be more expressed in human lung tissue (the physiological target of infection) as compared to other 28 human tissues analyzed [58].

Of note, out of the 332 identified human proteins, 66 were targetable by 29 FDA approved drugs, by 12 candidates in clinical trials and by 28 compounds in preclinical phase. The identification of the drugs modulating the 332 human proteins that interact with viral partners in HEK-293 cells (MiST ≥ 0.70) was conducted both through a chemo-informatics analysis of open-source chemical databases and also using a literature search specific for target and pathway (Table 2).

These repurposing drugs might be promptly used to treat COVID- patients, shortening the path for regulatory approval. Additionally, the strategy of drug repurposing would also reduce the costs required for the discovery of a de novo designed drug. Interestingly, among these compounds, zotatifin, ternatin-4, and PS3061, belonging to the class of mRNA translation inhibitors and others approved drugs such as clemastine, cloperastine, siramesine and PB28 well known as SigmaR1/R2 ligands, displayed antiviral activity against SARS-CoV-2 (Table 2). Of note, zotatifin and PB28 showed very high inhibitory efficacy in TCID_50_ viral titer assay with IC_90_ values of 0.037 μM and 0.278 μM, respectively. Interestingly, in this assay, PB28 resulted ~20 times more potent than hydroxychloroquine (IC_90_ 5.78 μM), which, in fact, has been recently demonstrated to have little or no effect on hospitalized COVID-19 patients [59].

Another important feature of this study is the possibility to assay a combination of these potential drugs, which targets cellular interactors, with drugs that specifically target viral enzymes, thus leading to a more effective therapeutic strategy to treat COVID-19.

Although AP-MS does not distinguish direct and indirect virus–host interactions (see also Figure 1), the interaction map proposed by the authors may help to understand several aspects of SARS-CoV-2 biology and can be used to suggest new therapeutic treatments and prevention interventions.

Of note, in a recent proteomic study on SARS-CoV-2 Vero E6 cells infectome, 56 of the protein interactors identified by Gordon and colleagues showed an altered concentration after virus infection [60]. Among them, the NADH-cytochrome reductase CYB5R3, an interactor of Nsp7, is particularly interesting, as inhibitors have been generated for this protein in preclinical phase [61], opening the possibility to rapidly test them as antivirals.

Finally, it is worth mentioning that the data generated by Gordon and colleagues [17] have been recently used to integrate CoVex (CoronaVirus Explorer) databases [62]. CoVex is an interactive and user-friendly web platform that uses published datasets from both SARS-CoV-1 and SARS-CoV-2 interactome maps, integrating them not only with the human interactome but also with drug information with the purpose to identify novel drug candidates. In fact, the rationale for the development of CoVex is that viral interactions with host targets have cascading effects; therefore, human proteins that do not interact directly with viral gene products may still be valuable therapeutic targets. Such a platform is made available to biomedical and clinical researchers and has the advantage of allowing access to network medicine algorithms for both in depth data mining and also hypothesis testing, all in an interactive and user-friendly manner. It has to be appreciated that the authors warned clearly that, while CoVex may be a mighty tool for SARS-CoV-2 candidate drug hunting, every result obtained using the platform should be taken with caution and that potential drug candidates identified by the platform may not only fail as antiviral, but could even have a proviral effect [62]. One of such examples is the polyA binding protein PABPC1, whose downregulation increases viral production [18], as discussed in more detail below.

**Table 2:** List of PPIs-identified main candidates for repurposing drugs to treat COVID-19 patients.

References	Bait	Prey (Gene Name) or Process	Compound	In Vitro Evidence ^(1)^	Compound Approval Status
Gordon et al. [17]	(2)	mRNA translation	PS3061	IC_50_ = 20–500 nM	Preclinical compound
			Ternatin-4	IC_50_ = 71 nM	Preclinical compound
			Zotatifin	IC_50_ = 1.5 nM	Drug in clinical trial
	E	*BRD2/4*	dBET6	IC_50_ = 14 nM	Preclinical compound
			MZ1	K_d_ = 120–228 nM	Preclinical compound
	M	*ATP6AP1/ATP6V1A*	Bafilomycin A1	IC_50_ = 100 nM	Preclinical compound
	N	*CSNK2A2*	Silmitasertib	IC_50_ = 1 nM	Drug in clinical trial
	Nsp6	*SIGMAR1*	Clemastine	Ki = 67 nM	FDA-approved
			Haloperidol	Ki = 2.91 nM	FDA-approved
			Hydroxychloroquine	Ki = 85 nM	FDA-approved
			PB28	Ki = 13 nM	Preclinical compound
			Siramesine	Ki = 17 nM	Drug in clinical trial
			Cloperastine	Ki = 20 nM	FDA-approved
		*ATP6AP1/ATP6V1A*	Bafilomycin A1	IC_50_ = 100 nM	Preclinical compound
	Nsp12	*RIPK1*	Ponatinib	IC_50_ = 12 nM	FDA-approved
	Nsp14	*IMPDH2*	Mycophenolic acid	IC_50_ = 20 nM	FDA-approved
	ORF9c	*SIGMAR2*	Clemastine	Ki = 15 nM	FDA-approved
			Haloperidol	Ki = 54.1 nM	FDA-approved
			Hydroxychloroquine	Ki = 772 nM	FDA-approved
			PB28	Ki = 13 nM	Preclinical compound
			Siramesine	Ki = 0.12 nM	Drug in clinical trial
			Cloperastine	Ki = 900 nM	FDA-approved
		*F2RL1*	AZ3451 (PAR2 negative allosteric modulator)	pK_d_ = 15	Preclinical compound
	ORF10	*VCP*	ML240	IC_50_ = 100 nM	Preclinical compound
Bouhaddou et al. [63]	(3)	*AXL*	Gilteritinib	IC_50_ = 0.807 μM	FDA-approved
	N/A	*MAPK11, MAPK14*	Ralimetinib	IC_50_ = 0.873 μM	Drug in clinical trial
	(4)	*MAPK13*	MAPK13-IN-1	IC_50_ = 4.63 μM	Preclinical compound
	N/A	*MAPK14*	ARRY-797	IC_50_= 0.913μM	Drug in clinical trial
	(4)	*MAPK14, MAPK11, MAPK12, MAPK13*	SB203580	IC_50_ = 4.76μM	Preclinical compound
	N	*CSNK2A1, CSNK2A2*	Silmitasertib	IC_50_ = 2.34 μM	Drug in clinical trial
	(5)	*PIKFYVE*	Apilimod	IC_50_ = 0.08 μM IC_50_ = 0.007 μM	Drug in clinical trial
	(6)	*CDK*	Dinaciclib	IC_50_ = 0.127 μM IC_50_ = 0.032 μM	Drug in clinical trial
Gordon et al. [18]	Nsp6	*SIGMAR1*	Fluphenazine	pIC_50_ = 6.46	FDA approved
			Chlorpromazine	pIC_50_ = 6.05	FDA approved
			Haloperidol	pIC_50_ = 5.684	FDA approved
			Clemastine	pIC_50_ = 6.264	FDA approved
			Meclizine	pIC_50_ = 5177	FDA approved
			Amodiaquine	pIC_50_ = 6.428	FDA approved
			Hydroxychloroquine	pIC_50_ = 6.062	FDA approved
			Chloroquine	pIC_50_ = 6.036	FDA approved
			Amiodarone	pIC_50_ = 6.779	FDA approved
			Tamoxifen	pIC_50_ = 6.563	FDA approved
			Triparanol	pIC_50_ = 6.439	FDA approved
			Clomiphene	pIC_50_ = 6.257	FDA approved
			Propranolol	pIC_50_ = 5.435	FDA approved
	Nsp7	*PTGES2*	Indomethacin	pIC_50_ = 4.258	FDA approved
	ORF9c	*SIGMAR2*	Fluphenazine	pIC_50_ = 6.46	FDA approved
			Chlorpromazine	pIC_50_ = 6.05	FDA approved
			Haloperidol	pIC_50_ = 5.684	FDA approved
			Clemastine	pIC_50_ = 6.264	FDA approved
			Meclizine	pIC_50_ = 5177	FDA approved
			Amodiaquine	pIC_50_ = 6.428	FDA approved
			Hydroxychloroquine	pIC_50_ = 6.062	FDA approved
			Chloroquine	pIC_50_ = 6.036	FDA approved
			Amiodarone	pIC_50_ = 6.779	FDA approved
			Tamoxifen	pIC_50_ = 6.563	FDA approved
			Triparanol	pIC_50_ = 6.439	FDA approved
			Clomiphene	pIC_50_ = 6.257	FDA approved
			Propranolol	pIC_50_ = 5.435	FDA approved
Stukalov et al. [21]	N/A	Inducers of DNA damage	Tirapazamine	2 µM ^(7)^	Drug in clinical trial
			Rabusertib	1 µM ^(7)^	Drug in clinical trial
	N/A	mTOR inhibitor	Rapamycin	1 µM ^(7)^	FDA-approved
	ORF3	*FLT3/AXL*	Gilteritinib	0.5 µM ^(7)^	FDA-approved
	(8)	*AKT*	Ipatasertib	5 µM ^(7)^	Drug in clinical trial
	N/A	Matrix metalloproteinase inhibitors	Prinomastat	2 µM ^(7)^	Drug in clinical trial
			Marimastat	2 µM ^(7)^	Drug in clinical trial

^(1)^ IC_50_ is the concentration of drug required for 50% inhibition of SARS-CoV-2 replication; Kd: equilibrium dissociation constant; K_i_: inhibitory constant; pK_d_ is the negative log of the K_d_; pIC_50_ is the negative log of the IC_50_. (2) In this case, the bait that started the process of identification of repurposing drugs was Nsp2, found to interact with eIF4E; two compounds (Tomivosertib and 4E1RCat) indirectly targeting eIF4E were found to be inactive [17]. As described in the text, next, the authors expanded testing on mRNA translation targets, in particular Zotatifin (targeting eIF4A), Ternatin-4 (targeting translation initiation) and PS3061 (targeting ER translocation). (3) An AXL-ORF3 high-confidence interaction was identified by Stukalov et al. [21]; a high-confidence interaction was identified between AXL and viral baits S, M, Nsp6, ORF3a, ORF7a and ORF7b by Samavarchi-Tehrani et al. [22]. (4) A MAPK13-ORF14 high-confidence interaction was identified by Laurent et al. [24]. (5) An high-confidence interaction was identified between PIKFYVE and viral baits S, Nsp2, Nsp4, Nsp7, Nsp10, Nsp12, Nsp16 by Laurent et al. [24]; an high-confidence interaction was identified between PIKFYVE and viral baits Nsp4, Nsp7, Nsp13, ORF3b and ORF7b by Samavarchi-Tehrani et al. [22]. (6) CDK4-Nsp10 and CDK12-Nsp15 high-confidence interactions were identified by Li et al. [20]; CDK5-Nsp16, CDK13-Nsp7 and CDK16-ORF3a high-confidence interactions were identified by Laurent et al. [24]; high-confidence interactions between CDK1 and viral baits Nsp2, Nsp4 and Nsp15, CDK5 and viral bait Nsp2, CDK9 and viral bait Nsp7, CDK12 and viral baits Nsp5, Nsp13, Nsp15 and Nsp16, CDK13 and viral baits Nsp7, Nsp13, Nsp15 and Nsp16 were identified by Samavarchi-Tehrani et al. [22]. ^(7)^ In the study by Stukalov et al. [21], treatments with significant inhibition effects on SARS-CoV-2 replication were reported. (8) AKT2-M and AKT2-S high-confidence interactions were identified by Laurent et al. [24]; an AKT1-Nsp2 high-confidence interaction was identified by Samavarchi-Tehrani et al. [22].

After defining the PPI map between SARS-CoV-2 and human proteins, the same group deepened their findings by publishing, just three months later, a second study (listing Nevan Krogan as “Lead Contact”) in which they performed a MS-based approach to analyze both protein abundance and global phosphorylation pattern of in SARS-CoV-2 infected Vero E6 cells [63]. Using nano-LC-ESI/Orbitrap and data-independent acquisition proteomic approach, in the first 24 h after infection they observed many more changes in the levels of phosphorylation rather than in protein abundance. In the subset of proteins that displayed a variation in concentration of the infection time course analyzed, only a limited number increased in concentration and, as expected, they were almost all viral proteins; on the other hand, out of the cellular proteins that did indeed change in concentration upon infection, the majority of them showed a decrease, consistent with the host mRNA export mechanism/translation shut off common to the life cycle of several viruses [64,65]. GO enrichment analysis revealed that, out of the cellular proteins that were downregulated, most are involved in platelet regulation, thrombosis and prevention of blood coagulation pathways, highlighting their potential role in stroke and blood coagulation observed in COVID-19 patients [66]. As mentioned above, quantitative MS-analysis over the course of infection revealed an increased number of significantly regulated phosphorylation proteins and sites, demonstrating that phosphorylation signaling represents a primary host response to SARS-CoV-2. In particular, the authors detected 25 phosphorylation sites in SARS-CoV-2 viral proteins that, combined with a proteomic dataset of Davidson and colleagues [67], resulted in a total of 49 sites on seven different viral proteins. It is expected that phosphorylation sites in membrane viral proteins (M protein, Nsp9 and N protein) could play key functional roles. Interestingly, out of the previously identified 332 human interactors (described above in the present review), the Krogan group found, in this second study, that 40 of them are differentially phosphorylated upon infection [63]. Of note, the SARS-CoV-2 N protein interacts with numerous RNA-processing proteins, which are differentially phosphorylated during infection, including LARP1 and RRP9. The significantly decreased phosphorylation level of LARP1 has been demonstrated to inhibit new cellular proteins [68], which could represent a SARS-CoV-2 strategy to prioritize synthesis of viral proteins over host proteins.

The authors also analyzed the global changes in kinase signaling and their effects on host protein phosphorylation. Based on the regulation of their known substrates, Bouhaddou and colleagues found changes in activity for 97 kinases, which are involved in signaling pathways that include p38/MAPK, AKT and ERK signaling, Rho GTPase and CK2 cytoskeleton signaling and cell cycle regulation [63]. Interestingly, the kinases whose activity is affected by SARS-CoV-2 infection are targeted by 87 drugs and compounds (10 FDA-approved, 53 undergoing clinical testing and 24 pre-clinical). The authors tested 68 of them at two different institutions (in New York and Paris) and in two cell lines (Vero E6 and A549-ACE2) and they observed that pharmacological inhibitors of p38 MAPK, CK2 signaling, PIKFYVE, and CDKs have strong antiviral efficacy.

To investigate SARS-CoV-2 dependence on the p38/MAPK pathway, SARS-CoV-2 infected ACE2-A549 cells were treated with the p38 inhibitor SB203580, and the resulting data showed not only a strong antiviral activity but also the inhibition of mRNA of the inflammatory cytokines IL-6, TNF-α and others (Table 2). These findings are relevant as the p38/MAPK pathway is involved in cellular response to environmental stress, pathogenic infection and pro-inflammatory cytokine stimulation [69,70]; moreover, immunological studies demonstrated an association between increased IL-6, IL-10 and TNF-α and lymphopenia with severe COVID-19 symptoms [71].

The authors investigated others pharmacological inhibitors of MAPK that were upregulated during infection (Table 2) and significant antiviral activity was observed for gilteritinib (AXL kinase inhibitor), ralimetinib (MAPK11 and MAPK14 inhibitor), MAPK13-IN-1 (MAPK13 inhibitor) and ARRY-797 (MAPK14 inhibitor).

Experimental data revealed also CK2 kinases as potential drug targets; in fact, CK2 is involved in regulation of stress granules pathways [17] and viral egress and dissemination (through CK2-mediated remodeling of the extracellular matrix) [63]. As expected, Silmitasertib (CSNK2A1 and CSNK2A2 inhibitor) was found to possess antiviral activity (Table 2). This finding well correlates with the evidence that CSNK2A2 has been identified as prey of viral bait N [17].

Another remarkable finding, emerging from the profiling analysis of kinase activity, was the reduction of CDK1/2 activities during infection, leading to the cell cycle arrest that may be beneficial for viral replication and progeny production. Accordingly, the use of dinaciclib (CDK inhibitor) evidenced strong antiviral activity (Table 2), confirming that the virus may enhance viral replication through regulation of cell cycle.

Finally, during infection the authors observed a significant regulation of phosphatidylinositol enzyme activities for PIK3CA, PLCB3 and PIKFYVE; in fact, apilimod (a PIKFYVE inhibitor) showed strong antiviral activity (Table 2) and the authors attributed this finding to a mechanism of regulation by phosphorylation of PIKFYVE upon viral infection.

More recently, the same group has extended this ongoing project comparing the viral-human protein–protein interactomes generated from the three different and highly pathogenic coronaviruses SARS-CoV-2, SARS-CoV-1 and MERS-CoV. With the same approach used in the previous study (AP-MS) and the same cellular system (HEK293T cells), they identified 366 and 296 high confidence interactions for SARS-CoV-1 and MERS-CoV, respectively. Additionally, they also identified 57 interactors of the SARS-CoV-2 Nsp16, not analyzed in their previous study obtaining a new integrated dataset containing (57 + 332) 389 interactions for SARS-CoV-2. The authors also performed the IF experiments in HeLa cells with the aim to localize individually expressed coronavirus proteins (Table 1). Localization analysis showed same cellular localization for the majority of orthologous coronavirus proteins, according to the notion that orthologous proteins show similar function. Furthermore, by using cellular compartment GO enrichment analysis, they compared the localization of the expressed viral proteins with the localization of their preys showing that in several cases the localization of the viral protein is in agreement with the localization of the interaction partners. An example is ER enrichment for interactions with Orf8 and the enrichment of the Nuclear Pore for Nsp9 host protein interactors.

Next, they quantitatively compared the virus-human interactions for each virus. This analysis demonstrated that viral structural proteins M and N and viral non-structural proteins Nsp7, Nsp8 and Nsp13 share the same interactions across the three coronaviruses, suggesting that they are involved in processes essential for the *Betacoronavirus* genus and, therefore, the identified host proteins may also be potential drug targets against future emerging pathogens of the *Betacoronavirus* genus. In particular, among the interactors of the N structural protein of the three viruses, the authors identified casein kinase II and RNA processing regulators (including the already mentioned polyA binding protein PABPC1 discussed below); among the interactors of the Nsp7 protein, the authors identified prostaglandin E synthase 2 (PGES-2) discussed below; among the interactors of Nsp8, they identified exosome and components of the translation machinery (ribosome biogenesis components and the signal recognition particle, 7SK snRNP); finally, among the interactors of the Nsp13 protein, they identified protein kinase A, centrosome (including PCNT), and Golgi organization (including GOLGA2); both PCNT and GOLGA2 will be discussed below.

On the other hand, some biological processes (such as intracellular transport regulation, heat response and proteasomal degradation) were hijacked by each coronavirus by interaction with different host proteins in the pathway. Similarly, in 51% of the cases, the same host protein was targeted by different (non-orthologous) viral proteins. For instance, human proteins targeted by MERS-CoV Orf5 were also found to interact with SARS-CoV-2 Orf3a; also, human proteins targeted by SARS-CoV-2 Nsp8 were also found to interact with MERS-CoV Orf4a (among which MPRS5). In the latter case, the authors identified some structural homology between the C-terminal region of SARS-CoV-2 Nsp8 and a predicted structural model of MERS-CoV Orf4a [72], thus furnishing an elegant molecular explanation to the experimental observation these two non-orthologous viral proteins bind the same human preys.

In order to validate the biological significance of the interactions identified by AP-MS, in this work, the authors inhibited the expression of 331 human interactors identified in their previous work [17], plus the ACE2 protein as a positive control, for a total of 332 proteins. The inhibition experiments were performed in ACE2-expressing A549 cells (A549-ACE2) by siRNA knockdown and in Caco-2 cells by CRISPR-based knockout. For each inhibition experiment, a fraction of the cells were monitored for inhibition efficiency and for viability, while the rest were infected with SARS-CoV-2 to assess the effect of the downregulation of the human interactor on virus replication. Analysis of perturbation efficiency demonstrated that siRNA knocked down at least 50% almost all the genes tested (93%) in A549-ACE2 cells and in 95% of the cases protein expression knock down had little or no effect (80% or more viability); as a consequence, the reported A549-ACE2 dataset includes 331 gene knockdowns. On the other hand, with CRISPR-based knockout an editing efficiency of at least 80% was observed for the vast majority (89%) of the 332 genes tested in Caco-2 cells; as a consequence, the reported Caco-2 dataset includes 286 gene knockdowns, due to efficient removal (equal or higher that 80%) of essential genes. Based on the effect of the host protein downregulation on virus replication, each interactor was classified as proviral dependency factor (whose inhibition significantly decreased virus production) or antiviral host factors with restrictive activity (whose inhibition significantly increased virus production). As expected, ACE2 downregulation strongly decreased virus production in both assays. In more detail, 31 dependency factors and 3 antiviral factors were identified by the experiments performed in A549-ACE2 cells, while 40 dependency factors and 4 antiviral factors were identified by the experiments performed in Caco-2 cells. Obviously, proviral dependency factors are particularly interesting as drug targets because inhibiting the interaction between the viral protein and the cellular factor (rather than reducing the expression of the host factor) may be a viable therapeutic option to prevent viral replication in vivo. Reduction of cellular protein expression of RAB2A, NGDN, SIGMAR1, ATP6AP1 and PPT1 significantly prevented virus replication in both experimental systems (A549-ACE2 and Caco-2 cells). Of these five host proteins, the same group previously studied in particular sigma-1 (non-opioid receptor sigma 1, encoded by the *SIGMAR1* gene) demonstrating, as described above, that the PB28 ligand of *SIGMAR1* gene product was a potent inhibitor of viral growth, with a 90% inhibitory concentration (IC90) = 0.278 μM, thus resulting ~20 times more potent than hydroxychloroquine (IC_90_ = 5.78 μM) in the same assay (Table 2) [17]. As described below, in the last part of the article the authors also examined the effects of potential ligands of sigma-1 on the outcome of COVID-19 patients [18]. Several of the host proteins identified as dependency factors in either one of the assays, among which the already mentioned prostaglandin E synthase 2 (PGES-2), were interactors of Nsp7 of SARS-CoV-1, SARS-CoV-2 and MERS-CoV; as described above, Nsp7 is one of the viral proteins which showed a very high fraction of shared interactions conserved across the three viruses. Other examples host dependency factors (whose inhibition significantly decreased virus production) found to interact with the same orthologous viral proteins were the already described PCNT and GOLGA2, interactors of Nsp13 of the three viruses. As discussed above, these cellular proteins may also be potential drug targets against future emerging pathogens of the *Betacoronavirus* genus. As might be expected, among conserved interactions the authors also found antiviral host factors with restrictive activity (whose inhibition significantly increased virus production). One of such factors is the already mentioned polyA binding protein PABPC1, which, as summarized above, was found to be one of the interactors of the N structural protein of the three viruses.

Next, the authors studied in deeper detail some of the host dependency factors identified downregulating or knocking out their expression. Among these, the mitochondrial import receptor subunit Tom70 (encoded by the gene *TOMM70*), interactor of both SARS-CoV-1 and SARS-CoV-2 Orf9b, is particularly interestingly because Tom70 plays a role in turning on the activity of the Mitochondrial AntiViral Signalling (MAVS) protein, one of the molecular pathways which induces apopotosis of a cell after virus infection [73,74]. Interestingly, an ORF9b-MAVS proximity interaction has been detected in all BioID core datasets here reviewed [22,23,24]. Moreover, Tom70 has also been shown to be involved in mounting an interferon response [75]. Indeed, not only both SARS-CoV-1 and SARS- CoV-2 Orf9b localized to the mitochondria both when overexpressed after transient transfection and in the context of physiological viral infection, but after transfection in HeLa cells they co-localized with Tom70 and the authors also observed a decrease in Tom70 expression after viral infection, already suggesting a potential mechanism of the virus to escape antiviral response. The authors confirmed the Orf9b-Tom70 interaction by co-immunoprecipitation and proposed a model of Orf9b interaction at the substrate binding site of Tom70 based on a 3 Å cryoEM structure they obtained. They also suggest how the phosphorylation of Orf9b, identified in a previous work by the same group already discussed in this review [63], may modulate the Orf9b-Tom70 interaction. Previous reports suggest that Tom70 interaction with cellular protein Hsp90 is essential for Tom70 function in the interferon pathway and for induction of apoptosis of a virus infected cell [74,76] and the authors suggest that Orf9b may interfere with the Tom70-Hsp90 interaction. Strangely enough, the authors themselves recognize that their “functional data, however, shows that Tom70 has at least some role in promoting infection rather than inhibiting it” [18]. Alternatively, as Tom70 is essential for import of PTEN induced kinase 1 (PINK1), the authors suggested that impairment of mitochondrial import efficiency caused by Orf9b binding to Tom70 substrate binding pocket may lead to the mitochondrial dysfunctions which is induced by the ORF9b of both viruses, as observed also in a different report [21] as described elsewhere in this review. If the exact role of the Tom70-Orf9b interaction in the viral life cycle still need more experimental evidence to be clearly elucidated, on the other hand, it is clear that the proteomic approaches described often point to the same cellular target (in this case Tom70) even if the experiments are performed by different groups [17,18,21,22,23,24].

In particular, they studied the outcome of COVID-19 patients coincidentally treated with FDA-approved therapeutics directed against two of the proviral dependency factors (whose inhibition significantly decreased virus production): the above-mentioned prostaglandin E synthase type 2 (PGES-2, encoded by *PTGES2*) and sigma non-opioid receptor 1 (sigma-1, encoded by *SIGMAR1*). It is worth reminding here that PGES-2 not only was proven to be a host dependency factor, but it was demonstrated to be an interactor of interactors of Nsp7 of SARS-CoV-1, SARS-CoV-2 and MERS-CoV, suggesting that it is involved in processes essential for the *Betacoronavirus* genus and, therefore, it may be a potential drug target even against future emerging pathogens of the *Betacoronavirus* genus [18]. Interestingly, PGES-2 is inhibited by indomethacin, an FDA-approved prescription NonSteroidal Anti-Inflammatory Drug (NSAID). By computational docking, the authors found that Nsp7 is predicted to bind PGES-2 adjacent to the enzyme indomethacin binding site. Only very high concentrations of indomethacin inhibited in vitro SARS-CoV-2 [18] and SARS-CoV-1, but the drug was effective against canine coronavirus in vivo [77], supporting the hypothesis that PGES-2 may also be a potential drug target against other members, even future emerging pathogens, of the *Coronaviridae* family. The human in vivo results of a non-interventional study were even more intriguing. In fact, the authors compared the outcome of 244 subjects, diagnosed with a SARS-CoV-2 infection who, by chance, initiated a course of indomethacin, with that of 474 SARS-CoV-2 positive subjects who, incidentally, initiated the prescription NSAID celecoxib (which does not target PGES-2). Interestingly, celecoxib users were more likely to require hospitalization than matched indomethacin users and, despite the small size of the sample, this is a nice example of mechanism-based drug discovery. Next, the authors focused on sigma-1, which, as stated above, proved to be a proviral dependency factor (whose inhibition significantly decreased virus production) in both assays and was found to be an interactor of Nsp6 of both SARS-CoV-1 and SARS-CoV-2 [18]; moreover, sigma-1 was identified as SARS-CoV-2 Nsp6 interactor also in other investigations (Section 3 below). In agreement with the previous findings, the authors demonstrated that amiodarone, a sigma ligand drug, inhibited the replication of both SARS-CoV-1 and SARS-CoV-2 Vero E6 cells (Table 2). Moreover, 13 FDA-approved drugs (including the abovementioned amiodarone) with nanomolar affinity for sigma receptors were all found by the authors inhibitors of SARS-CoV-2 in A549-ACE2 cells with IC_50_ values under 10 μM, although there was no evident correlation between antiviral activity and sigma receptor binding affinity. Among the 13 drugs, Fluphenazine, Chlorpromazine and Haloperidol were typical antipsychotics and therefore good candidates for real-world analysis, because users of typical anti-psychotics can be easily identified in a patient cohort using medical billing data. Again, the authors observed the outcome of subjects diagnosed with a SARS-CoV-2 infection (*n* = 1131) who were users of the three typical antipsychotics (which have in vitro antiviral effects and sigma binding activity) with SARS-CoV-2 positive subjects (*n* = 1516) who were users of five different atypical antipsychotics (which the authors demonstrated not to have antiviral activity against SARS-CoV-2 in vitro in A549-ACE2 cells and which were not predicted to bind sigma receptors). Very interestingly, twice as many users of atypical antipsychotics compared to users the sigma-ligand typical antipsychotics progressed to severe COVID-19, requiring mechanical ventilation. It has to be appreciated that Gordon and colleagues are very cautious in the interpretation of their results, underlining that “typical antipsychotics are known to bind to a multitude of cellular targets” [18]. However, (a) the observation that sigma-1 was found to be an interactor of SARS-CoV-2 Nsp6 in all the reports which searched for preys of this viral protein (see Section 3 below); (b) the evidence that sigma-1 proved to be a proviral dependency factor (whose inhibition significantly decreased virus production) in both assays used by Gordon and colleagues; (c) the significant size of the clinical sample (a total of 2647 patients examined) make these results very interesting and an even nicer example of mechanism-based drug discovery.

Li and colleagues conducted a study based on an integrated proteomics approach which can be summarized into three main parts [20]. In the first part of the study, they characterized the intra-viral interactome, through genome-wide yeast-two hybrid screens and co-immunoprecipitations experiments. Among the 28 SARS-CoV-2 gene products tested, the authors identified 58 intra-viral PPIs accountable for virus replication and which may be involved into immune evasion and viral pathogenesis. Of particular interest, an N-E and an E-ORF9b interactions were described, together with an M-N interaction. The potential significance of these intra-viral PPIs will be discussed in Section 2.2 below.

In the second part of the study, the authors generated the virus–host interaction map. With the same methodology used by Gordon et al. [17] (AP-MS see also Table 1) each SARS-CoV-2 protein fused with an N-terminal 3xFlag-epitope was expressed in HEK293 cells and after lysis, the affinity -purified host cellular partners were digested and analyzed by LC coupled to tandem MS. They identified 286 cellular preys interacting with SARS-CoV-2 baits to obtain 295 high-confidence interactions after filtering with MiST. Network analysis showed, among others, host targets related to inflammation and innate immune responses (Table 1), offering a possible explanation to the COVID-19 related respiratory symptoms. The interactors found in common between this interactome and that generated by the Krogan Lab were 45, with only the 16% of overlap. The interactors binding the same baits in common with the interactome generated by the Krogan Lab are shown in Table 3.

The limited number of common interactors is quite strange, also considering that with similar setting conditions, a more consistent overlap would be expected. Maybe, this could be due to the different tagging (see Table 1 for comparison) or lysis conditions used. Among common interactors the authors reported TRIM59, TBK1, G3BP1, G3BP2, RAE1, and SigmaR1 related to host innate immune responses to virus infection, however as reported in Table 3 of these only TRIM 59, G3BP1, G3BP2 and RAE1 showed the interaction with the same bait in both interactomes. Specifically, TRIM 59 interacts with ORF3a, G3BP1and G3BP2 interacts with N, RAE1 interacts with ORF6, in both interactomes (see Table 3); on the other hand, TBK1 interacts with ORF 6 in the interactome generated by Li et al. [20] while the same protein was found to interact with Nsp13 in the PPI map generated by Gordon et al. [17]. In the case of *SIGMAR1* gene product, Li et al. found it interacting with structural protein M, instead, all the other reports reviewed here found that Sigma R1 interacts with non-structural protein Nsp6 (see Table 3).

However, Li and colleagues found an important interaction (not captured neither by the Krogan lab nor by Stukalov and colleagues) between SARS-CoV-2 Nsp10 and NKRF which was further confirmed by co-immunoprecipitation experiments. The authors also observed that, in contrast to Nsp12, Nsp13, or Nsp15, which had little or no effect on IL-8 induction when individually expressed in lung epithelial A549 cells, Nsp10 instead facilitated IL-8 induction, suggesting that Nsp10 specifically promotes IL-8 induction. Interestingly, quantitative proteomic analysis that the authors carried out in the third part of the study showed up-regulation of IL-8 and IL-6 in peripheral blood mononuclear cells (PBMCs) isolated from severe COVID-19 patients in comparison to mild ones. Therefore, the proteomics experiments presented by the authors support the hypothesis that Nsp10, by sequestering NKRF and interfering with its repressing ability, contributes to increase both IL-8 and IL-6 expression. These overexpressed cytokines mediate chemotaxis of neutrophils, which, in turn, are responsible for the devastating host inflammatory response observed in severe COVID-19 patients. In conclusion, this study provides an integrated platform (virus interactome and proteomics analysis) able to elucidate mechanism of SARS-CoV-2 pathogenesis and to better clarify the pictures of clinical features related to innate immune response in COVID-19.

Davies and colleagues applied a MS-based workflow for interactome and proteome analysis enabling the rapid comparison of Nsp2- and Nsp4-host interactomes related to three different *betacoronaviruses*: SARS-CoV-1, SARS-CoV-2, and hCoV-OC43 [19]. This latter virus—an endemic species—is associated with the common cold and, consequently, with a different pathogenicity in comparison to the SARS strains. So far, no precise function is known for Nsp2, which is not conserved and it is even dispensable in SARS-CoV-1; on the other hand, the highly conserved transmembrane glycoprotein Nsp4 has a well-defined role in formation of replication complex associated double membrane vesicles. Therefore, according to the authors’ rationale, a comparative analysis of their interactome across these different systems might be useful not only to identify both conserved and unique interactors but also to uncover molecular mechanisms which play an essential role in the control of alterations of virulence. Specifically, they first performed quantitative comparative analysis between SARS-CoV-1 and SARS-CoV-2 Nsp2 interactors. In a second step of this study, a comparative profiling of Nsp4 interactions was performed also including hCoV-OC43 strain.

To generate the interaction maps, the authors used AP-MS and a tandem mass tag (TMT)-multiplexed quantitative proteomics approach. Briefly, the authors transfected HEK293T cells (also used in other reports here summarized, see Table 1) with constructs expressing FLAG-tagged Nsp2, Nsp4 and GFP (mock—used as control experiment in order to check nonspecific background protein during co-immunoprecipitation). The bait proteins together with their preys were then FLAG-immunoprecipitated and, through a bottom-up approach, the proteins were reduced, alkylated, digested, and before undergoing to LC-MS/MS analysis, tryptic peptides were preventively labeled with TMT for relative quantification of protein abundances. Then, proteins were identified, quantified and compared on the basis of TMT reporter ions intensities.

In relation to Nsp2, the authors identified 6 high-confidence interactors for SARS-CoV-2 and 11 for SARS-CoV-1. By using data from relative quantifications, they also found a group of host proteins clearly enriched for SARS-CoV-1(GIGYF2, PHB2, EIF4E2 and HDAC8) and others host proteins strongly enriched for SARS-CoV-2, such as FOXK1 and NR2F2. Interestingly, among the host proteins shared by both the two SARS-CoV strains, the authors found prohibitins (PHB and PHB2) and STOML2. These proteins together promote the activity of mitochondrial membranes; in fact, it was previously observed that upregulation of STOML2 is associated with an increased production of ATP and a reduction of apoptosis induction [78]. Therefore, the authors speculated that this is a conserved mechanism for the SARS strains used to ensure a pro-viral environment in the host cells. The authors also highlighted other known important evidences already reported on these interactors; among them, the role played by STOML2 interaction with hepatitis C virus in the stabilization of viral RNA replicase complexes [79] and the function of PHB in facilitating the entry of other viruses [80,81]. Such evidences suggest that these interactors might be important potential targets for a pan-RNA virus therapy.

Concerning Nsp4, 29 high-confidence interactors were identified for this bait in the case of SARS-CoV-2, 20 in the case of SARS-CoV-1 and 13 for OC43. The preys common to all the three interactomes are in large part ER-membrane associated proteins, while many specific Nsp4 interactors of SARS-CoV-1 and OC43 were found catalogued as nuclear-localized. The authors also found that several common SARS Nsp4 preys were involved in cellular communication, cell differentiation and cell death. In general, it was found that processes significantly enriched for SARS-CoV-1 (for example, ERAD pathway, ER mannose trimming and ubiquitin-dependent protein catabolic processes), were, instead, represented at a lower extent for SARS-CoV-2, and even less enriched for OC43.

As the same authors stated one limitation of this study is the use of transiently transfected viral proteins and, opposite to virus infection, this approach based on AP-MS provides the detection of only viral bait-host preys PPIs but not viral RNA-protein interactions, which could possibly affect the Nsp2 and Nsp4 interactome. However, it is important to underline that this is a common limitation inherent to the approaches used in the studies here reviewed, as will be discussed in the following sections. Despite this limitation, the comparative approach of the study has certainly the merit to better clarify the role played by these hCoV non-structural proteins in virus life cycle, shedding light on the potentially different mechanism of pathogenicity of OC43 compared to the two SARS strains.

Finally, as the same authors observed, no interactions were found in common to those generated by Gordon and colleagues. However, according to our inspection, an Nsp2- FOXK1 interaction is in common with the findings by Li and colleagues [20] and an Nsp4-HSPA5 interaction has been identified also by Stukalov and colleagues [21]; this latter interaction has been also confirmed by Laurent and colleagues [24] and by St. Germain and coworkers [23] in proximity labeling studies as described in Section 2.3 (see Table 3).

### 2.2. Mapping the SARS-CoV-2 Interactome Generated in A549 Lung Carcinoma Cells by AP-MS

Stukalov and colleagues performed another extensive AP-MS study by tagging (with a C-terminal HA tag) not only 24 SARS-CoV-2 proteins but also 27 proteins of the homologous SARS-CoV virus and even 1 protein (ORF3) of the HCoV-NL63 coronavirus and 2 proteins (ORF4 and ORF4a) of the HCoV-229E coronavirus [21]. The tagged proteins were expressed in A549 lung carcinoma cells by mean of lentiviral transduction (Table 1). Then, the authors applied AP-MS to discover the cellular interactors of the viral proteins. In particular, by this approach, they identified 1484 interactions between the 24 SARS-CoV-2 and 27 SARS-CoV baits and the A549 preys. The majority of the interactions (853) was shared by both coronavirus strains; however, a subset of them was found to be specific of either SARS-CoV-2 or SARS-CoV proteins. The identified interactions point to a large number of cellular pathways which are presumably affected by the viral gene products; in particular, specific viral proteins target cellular regulators involved in key homeostatic pathways, such as DNA damage response (ORF7a interacting with cellular ATM and ATR), innate immunity (ORF7b interacting with cellular UNC93B1 and MAVS) and, finally, stress response (N interacting with cellular HSPA1A). Interestingly, Li and colleagues [20] also identified an ORF7a-ATR interaction (Table 3). Among the 293 interactions that were SARS-CoV-2 specific, the authors described alterations that were exclusive to SARS-CoV-2; for instance, they demonstrated that only SARS-CoV-2 ORF8 binds to TGFB1 and TGFB2 and to the LTBP1 protein, while the same cellular preys have no interaction with none of the SARS-CoV ORF8 homologs.

To better evaluate the effects of the identified interactions on cellular metabolism, next the authors analyzed the perturbations induced in the cellular proteome by the individual expression of each of the 54 viral proteins listed above. This piece of data, together with the list of interactors of the respective viral protein, provided additional information about each viral protein function. Again, many of the observed perturbations were common to both viral strains gene products. For instance, ORF9b of both viral strains induced mitochondrial dysfunctions, in agreement with the observation that both proteins interact with the mitochondrial protein Tom70. Indeed, a SARS-CoV-2 ORF9b-Tom70 interaction has also been detected not only by Gordon and colleagues [17] but also in all BioID studies here reviewed [22,23,24], as summarized in Table 3. However, in line with the observation that 293 of the detected interactions were SARS-CoV-2 specific, the authors described alterations which were exclusive of SARS-CoV-2, for instance upregulation of the cellular proteins involved in the cholesterol biosynthetic process, which occurred only after expression of the SARS-CoV-2 Nsp6 protein and not after expression of the SARS-CoV homolog.

Next, the authors infected ACE2 stably transfected A549 human cells with SARS-CoV-2 and assessed, in a time dependent manner, the effect of viral infection on the host transcriptome, proteome, ubiquitinome and phosphoproteome. In agreement with already published data [82,83], transcriptomic analysis demonstrated no significant upregulation of the interferon family genes (for instance *IFNB* and *IFIT3*), suggesting virus mediated shut off of this antiviral defense system. On the contrary, the mRNAs of both NF-κB and stress response gene (such as IL-6, CXCL2, JUNs and JUNB) significantly increased in a time dependent manner after virus infection. The changes observed at transcriptomic level were paralleled by the data obtained at the proteomic level. In fact, the authors identified 1053 regulated proteins but, in agreement with the transcriptomic data, no significant interferon response pattern was detected, suggesting again that this is one of the strategies evolved by the virus to escape cellular defense mechanisms. The proteomic data were then integrated with a global analysis of the ubiquitination pattern following SARS-CoV-2 infection, which highlighted changes of ubiquitination in 884 sites. In fact, integration of the proteome and ubiquitinome data allowed the detection of concomitant ubiquitination and decreased concentration for a number of cellular proteins involved, among other processes, in caveolar-mediated endocytosis. In particular, the authors list POLR2B, DHFR, EFNB1 and TYMS as relevant examples of proteins which were ubiquitinated and whose concentration decreased following viral infection. Additionally, the same SARS-CoV-2 ACE2 receptor was ubiquitinated on two lysines, at position 625 (previously identified) and at position 702 (identified for the first time), suggesting a mechanism of ACE2 degradation following SARS-CoV-2 infection. Moreover, several so far unidentified ubiquitination sites were detected also on viral proteins and these findings well correlated with the AP-MS identified interactions of viral baits and cellular E3 ligases (viral ORF3 with cellular TRIM47, WWP1/2 and STUB1; viral M and with cellular TRIM7; viral Nsp13 with cellular RING1), suggesting that the virus has evolved mechanisms to utilize the cellular ubiquitination apparatus to regulate its own life cycle. Finally, the authors also dissected the phosphoproteome of infected cells, quantifying the impressive number of 11,847 phosphorylation sites, of which 1483 were significantly altered upon SARS-CoV-2 infection. Concerning viral gene products, this analysis led to the identification of newly identified phosphorylation sites on ORF9b and on structural M, N and S SARS-CoV-2 gene products, phosphorylation occurring on sites known to be target of cellular kinases ERKs, GPCR, CSNKs, CAMKs, AKT and GSK3. However, of the listed kinases, only GSK3a was clearly reported to interact with viral proteins N (one of the putative targets) and ORF3, but no direct interaction with ORF9b were reported; the link between cellular kinase GSK3a and its putative viral target ORF9b might be through viral N protein, which, in another article reviewed here [20], has been demonstrated to directly interact with viral E, which, in turn, interacts with ORF9b. The hypothesis proposed by Stukalov and colleagues is at least in part supported by data that appears in other articles reviewed here. For instance, Gordon and colleagues indeed detected a direct interaction between viral structural protein N and the two Casein Kinases II CSNK2B and CSNK2A2 [17]. The theoretical discrepancy of the apparent lack of interactions between the other cellular kinases and their putative viral target might be explained hypothesizing transient, fast binding of the enzymes with their targets, which may easily escape detection via AP-MS as explained elsewhere in this review. Indeed, Laurent and colleagues, by using the proximity assay, as reviewed in this article, not only confirmed the already reported interaction between cellular GSK3a and viral N already detected by Stukalov and colleagues, but also revealed interactions between cellular CSNK1G3 and viral proteins M and S between cellular GSK3a and viral N and, finally, between cellular AKT2 and viral S and M [24]. Interestingly, the interactions listed above were not detected by all the groups which used AP-MS [17,20,21]. Concerning cellular proteins, the altered phosphorylation pattern suggests that viral infection affects the activity of the relevant role of kinases that regulate fundamental biochemical pathways, such as cell cycle progression (CDKs), DNA damage response (ATM and CHEK1/3), focal adhesion (EPHA2), stress responses (p38, JNK and ERK), cell survival (RPS6Ks) and, finally, cell growth, survival and motility (AKT). Interestingly, virus infection affected not only phosphorylation but also ubiquitination of YAP1, a member of the Hippo signaling pathway, which is an evolutionarily conserved signaling pathway involved in self-renewal, tissue regeneration, organ size control and, more recently, also angiogenesis [84].

In conclusion, a multi-level approach, based on PPI detected by AP-MS and on the simultaneous analysis of the host transcriptome, proteome, ubiquitinome and phosphoproteome following viral infections, allowed the authors to gain insights on the molecular mechanisms used by SARS-CoV-2 to progress through its life cycle, thereby causing as side effect same of the observed pathogenic effects. For instance, a molecular mechanism was proposed to explain the fibrosis observed in COVID-19 patients. In fact, the authors not only reported the interactions between ORF8 and the TGFβ factors (TGFB1, TGFB2 and LTBP1) and also between ORF3 and the TGFβ receptor components TGFR1 and TGFR2, but they also demonstrated upregulation of the TGFβ pathway, which explains the increase in fibrinogens and fibronectin observed by the authors in SARS-CoV-2 infected A549-ACE2 cells. It should be stressed that only SARS-CoV-2 ORF8, but none of the SARS-CoV-2 ORF8 homologs, binds to TGFB1 and TGFB2 and to the LTBP1 protein (see above).

Finally, the identification of several cellular pathways directly or indirectly affected by viral proteins allowed the authors to perform a mechanism-based drug screening. Among others, they showed that Rapamycin, an inhibitor of the mTOR kinase (which is activated by RHBE, a cellular protein overexpressed in SARS-CoV-2 infected cells) inhibited viral life cycle. Among the tested drugs, Ipatasertib (an AKT inhibitor) effectively prevented SARS-CoV-2 replication with apparently modest side effects as far as cell growth was concerned. The authors stressed that they “identified AKT as a potential kinase phosphorylating SARS-CoV-2 protein N” [21] although, as highlighted above, they detect no direct interaction between cellular AKT kinase and viral protein N by AP-MS. However, as mentioned above, an interaction between AKT2 and viral M was indeed detected by Laurent et al. [24] by means of proximity assay (instead of AP-MS) and a direct interaction between viral M and N was detected by Li and co-workers [20], thus furnishing a physical connection between AKT2 and viral N. This latter example is a straightforward demonstration of how the knowledge gained in the studies here reviewed can drive mechanism-based drug discovery.

### 2.3. Mapping the SARS-CoV-2 Interactome Generated in HEK293 Cells by BioID

A total of nine of the 29 SARS-CoV-2 proteins have one or more predicted transmembrane domains and, interestingly, the above-described studies based on AP-MS identified relatively few cellular interactors for this subgroup of proteins. With the aim to provide more specific and in-depth data on virus–host interactions which might escape detection by AP (for example, interactions within low solubility complexes/compartments, weak affinity interactions or transient interactions like enzyme-substrates in the case of high turnover rate enzymes), three studies were performed applying proximity-dependent biotinylation (BioID) [22,23,24]. In fact, as already highlighted in the Section 1.2, while AP-MS is well-suited for the identification of biochemically stable protein complexes, this procedure is less suited to identify neither weak or transient interaction (which may easily break apart during cell lysis prior to affinity purification, see Figure 1) nor even stable interaction occurring in insoluble cellular compartments (such as membranes or chromatin), necessitating very harsh extraction conditions in order to extract the protein content including transmembrane proteins (conditions which would disrupt even strong, stable non-covalent interactions prior to affinity purification).

In the study by Laurent et al. [24], the proximity labeling BioID assay with BirA* enzyme was performed in HEK293 cells (Table 1). The experiments conducted on the 28 viral baits uncovered 10,267 high confidence interactions by a total of 2598 host binding partners. The dataset was then subjected to a rigorous filtering, resulting in 2128 unique cellular targets. Furthermore, with the aim to make data exploitation easier, this group provided an innovative 3D web interface (http://www.sars-cov-2-interactome.org/) (Table 1).

Importantly, this group carried out two set of experiments both in basal conditions and in the presence of polyinosinic-polycytidylic acid [poly(I:C)]. [poly(I:C)], which is a synthetic *ds*RNA analogue, stimulating a rapid cellular response, similar to that occurring upon viral infections. Therefore, according to the authors’ rationale, comparing the interaction map observed in basal conditions with that obtained in infection-mimicking conditions may help to better elucidate the complex network of interactions established by viral and host proteins expressed or activated upon virus entry into the cell. In particular, these parallel sets of experiments were very useful in identifying key response pathway components associated with specific viral bait, which could serve as potential targets to block virus life cycle. In fact, among others, the authors, while confirming the interaction of ORF9b with Tom70 also detected by AP-MS [17,21] (Table 3), also identify an ORF9b-MAVS interaction which was not detected by AP-MS studies. More interestingly, the authors found an interaction of ORF9b with ISG20 (Interferon Stimulated Gene 20kDa protein), which, importantly, was exclusively detected only following poly (I:C) pretreatment. Interestingly, a recent investigation demonstrated the inhibitory activity exerted by ISG20 (an IFN -induced 3′ -5′ exonuclease) against multiple viruses through upregulation of Type I Interferon Response Proteins [85]. Therefore, it was possible to speculate a role of Orf9 in targeting ISG20 and, as a consequence, subtracting it from its antiviral function. Therefore, this study not only confirmed the central role of ORF9b in blocking the innate immune host response targeting the antiviral signal transduction through its interaction with MAVS, but it also highlights its role in interfering downstream of the IFN signaling pathway through ISG20.

Another important evidence supported by the global analysis of proximity interaction maps data generated by ORF6 was its wide range involvement to counteract the host cell proper innate immune response. In fact, the authors found that ORF6 was unique, among all the 28 studied viral baits, in establishing proximal interactions with several nucleopore complex components, among which four of the six subunits of the central transport channel (NUP35/54/62/214). Of these in particular, the interaction of ORF6 with NUP62 and NUP214 may possibly interfere with nuclear import of transcription factors activating genes encoding proteins part of the innate immune response program as already found in the case of SARS-CoV-1 ORF6 [57,86]. Additionally, ORF6 was also the only bait detected to interact with the CCC complex components that can target NF κB for degradation. In light of these data, therefore, the authors speculated that ORF6 could impair host cell proper innate immune response through these important interactions.

Interestingly, in this study, the authors also identified ORF7a and ORF7b protein proximal partners potentially associated to anosmia and ageusia symptoms. In fact, among the ORF7a binding partners they identified REEP proteins, which are associated with membrane receptors and/or olfactory receptors regulation mechanisms [87]. In the case of ORF7b, its interaction with the glutamate receptor ionotropic delta-1 GRID1 [88] and its interaction with ROBO2 a protein involved in the development of olfactory bulb neuron [89] might explain the anosmia symptom in COVID-19.

Interestingly, among N host high-confidence interactors, the authors found G3BP1, G3BP2, CAVIN1, GSK3A/B and PRKRA. As the authors observed, N viral protein, due to its high abundance within the infecting virion, is the first protein released in the host cell even before the onset of viral RNA translation and, as a consequence, it might possess pro-viral function providing a cellular environment appropriate for virus replication. Therefore, the authors, continuing on this line, speculated that N might possibly localize inside the stress granules; in fact, G3BP1 and G3BP2 are components of these cytosolic organelles deputed to mRNA storage. Furthermore, the interaction with CAVIN1 also suggests that N might interact with caveolae formation. Of note, G3BP1, G3BP2 and CAVIN1 are interactors also found by AP-MS approach (Table 3) in the other studies here described [17,20,21].

Based on the analysis of specific interactomes obtained following poly(I:C) treatment, this study has the great merit in highlighting the main role played by ORF6 and ORF9b (outlined in this review) and also by N and Nsp2 viral baits in prejudicing the innate immune response observed in COVID-19 patients. While for only four viral proteins (Nsp5, Nsp8, ORF8 and ORF10) this approach led to limited number of results, much of the evidence about specific subcellular locations and pathways assigned to viral proteins are well and clearly supported by data generated in this study.

In another study, St-Germain et al. performed the BirA*-based BioID approach on a more limited number of SARS-CoV-2 virus proteins [23]. Specifically, 14 viral gene products were used: the nine encoding proteins predicted to possess at least one transmembrane domains (S, E, M, ORF3a, ORF7a, ORF7b, Nsp3, Nsp4 and Nsp6) and those coding for the less known (as judged by the authors) Nsp2, ORF3b, ORF6, ORF8 and ORF9b viral proteins (Table 4).

Briefly, the viral proteins were fused with an N-terminal BirA* coding region, and the fusion proteins were individually expressed in HEK 293 Flp-In T-REx cells. By this approach they uncovered 3566 proximity interactions with 1010 proximity host interactors. Interactome analysis revealed an extensive enrichment of SARS-CoV-2 proteins with host membrane proteins (plasma membranes, ER/nuclear, Golgi and ER-Golgi trafficking vesicles). Molecular functions significantly enriched include lipid transporter activity, SNARE binding and sterol binding activities. Interestingly, the authors observed high confidence interactions between SARS-CoV-2 proteins with both lipid/cholesterol transfer and membrane contact sites proteins. Starting from this observation, they speculated that SARS-CoV-2 might exploit the phosphatidylinositol 4- phosphate -driven cholesterol transport (system normally used at ER membrane contact sites, via recruitment of OSBP/OSBPL proteins) to generate the RO replication organelles membrane or membrane-bound “virus factories” [90,91,92,93], essential for scaffolding and protecting virus replication from host defenses [94]. Therefore, they highlight the importance of the human membrane contact site lipid transfer system and suggest it as a potential drug target in the treatment of COVID-19.

This study (in agreement with the above referenced study by Laurent et al. [24]) evidenced the significantly enriched interactome of SARS-CoV-2 ORF9b in protein of mitochondrial origin such as MAVS. This protein plays an important role in cell-based innate immune signaling. In fact, pathogen-associated molecular patterns (PAMPs, e.g., viral RNAs) are recognized by cellular pattern recognition receptors (PRRs) and the interaction between the complex PAMP-PRRs with MAVS activates both Type I interferon and NF-κB signaling pathways [95]. Since many viruses overcome these mechanisms by disrupting the MAVS activated signaling, a potential strategy for therapeutic treatment may be preventing the virus to interfere with MAVS signaling. In fact, as recently observed in the study of Jiang et al. [75], SARS-CoV-2 ORF9b suppresses MAVS-mediated type I IFN signaling by its binding with Tom70 (encoded by the *TOMM70* gene), which was detected also in this study as a major component of the ORF9bB interactome. Therefore, a possible strategy to contrast the virus response to the host defense may be to prevent the interaction of ORF9b-Tom70. Tom70 was found also as interactors of ORF9b in the AP-MS studies previously described [17,21] and, as reported in Table 3, was found also by the other investigations carried-out by BioID [22,23,24]. Another interesting result of this study was the enrichment of the ORF6 interactome with the nuclear pore complex (NPC) components. Interestingly an interaction between ORF6 and the nuclear pore component RAE1 has been confirmed in a high proportion of the studies here reported (Table 3).

### 2.4. Mapping the SARS-CoV-2 Interactome Generated in A549 Cells by BioID

In order to overcome the relatively low catalytic activity of the classical BirA* enzyme used in the original proximity labeling experiments and in the studies summarized above [23,24] Samavarchi-Tehrani and colleagues, used the new “miniTurbo” BioID assay in which BirA* enzyme is replaced by a N-terminally truncated BirA protein containing a dozen mutations, with an improved catalytic activity enabling efficient biotinylation within minutes [41] Specifically, in this study by the mean of a lentiviral delivery system, they performed the “miniTurbo” BioID assay on 27 SARS-CoV-2 viral proteins (Table 4) in A549 lung alveolar epithelial cells tagging in parallel both the viral proteins (on N- and C-terminus) and 17 cellular proteins as organellar markers (Table 1). Dataset obtained after profiling by BioID and by MS analysis was filtered by SAINT obtaining 7810 high-confidence proximity interactions of the viral baits with a total of 2242 human host proteins. In particular, the data showed a median of 205 proximity interactions for each viral bait and the hosts mostly localized to membranous compartments. As the same authors stated, the main challenge in this approach is due to the large datasets generated, particularly for baits localized to membranes, which uncovered hundreds of interactors and the consequent interpretation of their biological significance. Therefore, in order to better assess data analysis, they filtered these large host datasets by using humancellmap.org resource, thus selecting host interactors at different subcellular localizations. Interestingly, in this approach, the analysis of the proximity host proteins together with the localization of each viral proteins, can be used to deduce specific interactions at the basis of their functions. For example, the authors found ribosomal components as host interactors of viral proteins M, Nsp2, Nsp5, Nsp13, Nsp14 and ORF3a, most likely due to baits localization to the ER, while N, Nsp1, Nsp3, Nsp9 and Nsp16 were found to be related with cytosolic RNA-binding proteins or their interaction partners. These findings may suggest essential indications about the mechanisms used by SARS-CoV-2 to assume the control of host protein synthesis machinery, in particular, as already determined for several other viruses [96], by shutoff of host protein synthesis. Of note, interactions between Nsp1 and host translation initiation factors (in particular subunits of eIF3) suggest a role for Nsp1 in host protein synthesis shut off. In agreement with this findings Gordon and colleagues [17] demonstrated that compounds like Zotatifin and Ternatin-4, which interfere with translation initiation have antiviral activity (Table 2).

For the viral proteins Nsp7 and Nsp8, both BioID compartment enrichment analysis and immunofluorescence did not display particularly informative localizations. However, a mild enrichment associated with the regulation of cell cycle derived from the joint analysis of Nsp7/8 proximity host proteins was found using GO. Among these proximity interactors, the authors identified proteins linked to the DNA damage checkpoint, centrosome, histone binding, proteasome components E1 and E3 ubiquitin system proteins (Table 1).

The analysis of the proximity interactome of both Nsp4 and Nsp6 suggested a possible role of these viral proteins in the formation of viral replication organelles and membrane reorganization.

ORF9b was localized in the mitochondria showing its unique interaction landscape characterized by mitochondrial proteins. In fact, ORF9b was found to be in proximity of several outer mitochondrial membrane proteins, among which Tom70 (encoded by the *TOMM70* gene). Interestingly, the Tom70-ORF9b interaction was detected in all the studies here reviewed (Table 3). Of note, a proximity interaction between ORF9b and the MAVS protein was also detected by the other two BioID investigations here reviewed [23,24].

The authors performed a comparison with the previous AP-MS published data from the Krogan’s group, which showed an overlap of only 10% of the PPI for 13 different viral proteins. The authors explain this apparently limited overlap mentioning the *“standard, gentle lysis conditions”* used in AP-MS as *“not optimal for the capture of labile protein-protein interactions, or proteins associated with membranes”.* Moreover, they also observed that the occurrence of post-lysis artefactual interactions resulting *“from the extreme overexpression of transient transfection”, “can also explain some of the interactions captured by published AP-MS studies”* are not detected by the BioID mini Turbo approach. In addition, some interactions detected by AP-MS might have been missed by the BioID mini Turbo approach due to absence in the prey of solvent-exposed lysines or to insufficient time to allow biotinylation by the miniTurbo enzyme. Finally, differences in the biological system used (Table 1) might also contribute to explain the apparently limited overlap.

These Bio-ID MS data were also integrated by immunofluorescence microscopy experiments thus furnishing the subcellular localization of the SARS-CoV-2 proteins. In particular, the results show the localization of Nsp4, E, and ORF8 in the ER, ORF3b and ORF7a in the Golgi while Nsp6, S, ORF3a, M, ORF6, ORF7b were detected in both compartments. In general, these results were in agreement with other published data on viral proteins localization in the subcellular compartments, suggesting that the mini-Turbo labeling does not affect their localization. All the datasets generated in this study on the virus–host interactome are available at the website covid19interactome.org.

## 3. Discussion

### 3.1. Common Host Interactors Across Core PPI Datasets

A comparison of the results described in the various reports summarized in this review is not at all straightforward because, besides the different experimental approaches, some groups analyzed the interactions of almost all the viral proteins, while other groups analyzed only a subset of them (Table 4). If a viral protein–cellular host interaction is not artefactual, it is expected that such interaction should be detected by different groups, regardless of the experimental system used, at least when the experiments were performed in the same cells (Table 1). Moreover, as detailed in the first part of this review, it is expected that the AP-MS approaches should detect only relatively stable complexes, whereas proximity labeling approaches are expected to also detect more transient PPIs. Starting with this rationale, for each viral protein the cellular interactors identified in all the reports using an AP-MS approach have been compared, in order to identify cellular baits detected in at least two different reports [17,18,19,20,21]. The output of such comparison is illustrated in the left part of Table 3: the table lists, for each viral protein, which reports have studied such bait and, for each report, which cellular interactors have been captured by the same bait in at least another manuscript based on AP-MS. Next, we verified if these common interactors were also detected in at least one of the reports that analyzed viral proteins by a proximity labeling approach [22,23,24]. All the preys that matched the abovementioned criteria are listed in the right column of Table 3. It should be considered that, if a cellular interactor has been detected in at least three different reports by using two different technologies (AP-MS and proximity labeling), the possibilities that such interaction is artefactual are strongly reduced and it is, therefore, more likely that the detected interaction may indeed play a role in the viral life cycle. As described above, the Krogan group analyzed the 332 human interactors they identified [17] both as targets of existing drugs (already approved or in development: [17]) and in terms of biological significance, by perturbing their expression [18]. Among the proteins listed in the right column of Table 3, 20 were identified as interactors by the Krogan group [17,18]: interestingly, over half of them (the 12 highlighted in light-orange) were demonstrated to be either drug targets, or to be part of a complex/process targeted by drug candidates [17] or were host dependency factor [18]. The 11 cellular preys highlighted in grey in the right column of Table 3 have not been tested by the Krogan group simply because they were not identified as interactors by those authors [17,18]. However, these cellular proteins may be very promising drug targets for the reasons listed above.

### 3.2. Challenges and Limitations of These Approaches

According to the reported investigations, all the SARS-CoV-2-host interaction maps have been generated or in human embryonic kidney HEK-293T primary cells or in human lung epithelial A-459 respiratory cell lines (Table 1). Since SARS-CoV-2 can infect several human cell types the PPIs from HEK-293T or A-459 cells might miss tissue-specific factors, introducing biases [97]. On the other hand, even not exactly describing a SARS-CoV-2 physio-pathological model, the “workhorse” 293 cell system however expresses a wide panel of proteins and therefore it may be considered excellent bioreactor to identify possible interactions in living cells [23,24]. Moreover, Gordon and colleagues [17] pointed out that the interactors identified in HEK-293T cells were found to be more expressed in human lung tissue (the physiological target of infection) as compared to other 28 human tissues analyzed [58].

Continuing on this issue, both AP-MS and BioID have the inherent limitation to study one at the time the interactions between the viral bait and hosts proteins. As reported in the previous sections describing the protocols used in the studies reviewed here, both AP and BioID tools are, in fact, based on the expression of a single virus protein (see also Figure 1) and, therefore, do not really mimic what occurs in living cells during virus replication and spread. The absence, in these minimal systems, of cooperative virus protein interactions as well as of viral RNA, required by several viral proteins to correctly perform their function, has addressed several research groups either to conduct parallel experiments also in infected cells [24] or to support their “basal” interactomic datasets by additional orthogonal proteomics experiments on infected cells [18,21,63]. In any case, the studies here reviewed have convincingly demonstrated the ability of both AP-MS and BioID to identify large core datasets of high confidence host interactions regarding SARS-CoV-2 as well as the related SARS-CoV-1 strain and other viruses of the *Coronaviridae* family.

Another important point demonstrated here is the perfect complementarity of both the AP-MS -generated interactome and the proximity BioID interactome in supporting experimental evidence emerged from -omics experiments. Such an example are ubiquitination sites detected on viral proteins correlated well with the AP-MS-identified interactions of viral baits and cellular E3 ligases [21], or transient PPIs, like kinase-substrate interactions, not found by AP-MS but instead by BioID studies (see for examples notes of Table 2) which, in turn, provide an elegant explanation to observations found by phoshoproteomics experiments [21,63].

## 4. Conclusions

The studies described here provide a useful tool to understand and to interpret important signaling pathways at the virus–host interplay, suggesting preferential avenues in targeted anti-viral therapies. The SARS-CoV-2 interactome has been uncovered within two different cellular host machineries (HEK293 and A549 cells), resulting in a limited overlay in terms of common interactors as emerged by overlapping analysis performed by our group herein (Table 3). On the other hand, although limited in number, these common interactors that have been evidenced from our analysis in Table 3 might reveal important pathways considering that have been also corroborated by both the two different approaches (AP-MS and BioID). One such example is the interaction between Nsp6 and Sigma-1 (encoded by *SIGMAR1* gene), which led to the identification, among others, of Fluphenazine, Chlorpromazine and Haloperidol (Table 5), proposed as potential drug candidates for repurposing against COVID-19 [18].

Continuing on this point, another druggable target has been identified in the AKT kinase, a potential N viral protein interactor (via viral protein M [20,24] as described above) proposed to phosphorylate the same viral protein N [21]; in fact, the AKT inhibitor Ipatasertib was demonstrated to effectively prevent in vitro SARS-CoV-2 replication (Table 2 and Table 5) with modest side effect on cell growth [21].

According to our investigation on PPI datasets overlay, summarized in Table 3, additional examples that may be potential druggable targets are the host interactors identified in several studies, reported with the gene names in the Table 3 and highlighted in grey: *ATG9A, CAVIN1, CPD, HMOX2, RAB13, RAB14, TBL2, CNNM3, HSPA5, HERC1, CCDC22*. For instance, the *CAVIN1* gene encodes for the cavin-1 protein, which, together with caveolin-1, is among the major structural components of caveolae, and it has been demonstrated that cavin-1 and caveolin-1 play an important role in the respiratory syncytial virus life cycle [98]. Moreover, a recent study revealed that cavin-1 deficiency affects a number of coronary and myocardial functions, including ischaemic tolerance and stretch responses [99]. Interestingly, cardiac injury has been reported in COVID-19 patients [100], even in the absence of coronary arteries obstruction [101], therefore unlikely to be caused by altered coagulation observed in these patients [102]. Furthermore, a mechanism of entry of SARS-CoV-2 in cardiomyocytes has been recently proposed [103]. In light of these evidences, should the cardiac injury, observed in COVID-19 patients, be proven to be due, at least in part, to cavinin-1 sequestration by viral N, then inhibitors of the N-cavinin1 interaction may be valuable drug candidates.

This review has highlighted how, taken together, the core datasets from these studies might add more pieces to the intricate puzzle of mechanisms used by SARS-CoV -2 to hijack the cell’s machinery. One such example of dataset integration are the phosphoproteomics studies by Stukalov and colleagues [21], which brought to the hypothesis that viral proteins ORF9b, M, N and S were phosphorylated by the cellular kinases ERKs, GPCR, CSNKs, CAMKs, AKT and GSK3. Although only a GSK3-N interaction was detected in that investigation [21], additional supporting data came from the work of Gordon and colleagues who detected CSNK2-N interaction [17] and by the proximity assays of Laurent and co-workers [24] who detected interactions between cellular CSNK1G3 and viral proteins M and S, between cellular GSK3 and viral N and, finally, between cellular AKT2 and viral S and M.

We envisage that both intersection and combination of the different virus–host interactome datasets here reviewed can be helpful in a better understanding of SARS-CoV-2 pathobiology for developing effective antiviral therapies. In fact, while common shared interaction strengthen very high confidence drug targets, complementary results may increase functional and pathways coverage across different cell types generating a comprehensive interaction map.

These specific proteomics datasets here reviewed may inspire new studies aiming at better elucidating both specific and nonspecific host defense mechanisms against viral infection. An in depth interpretation of the complex SARS-CoV-2-host PPI maps based on high-throughput -omics studies here reviewed together with computational and chemo-informatics approaches as well as network medicine may provide a valid synergistic platform necessary for understanding virus–host–drug mechanisms and for predicting novel drug targets [62,104,105,106]. Interestingly, in this direction, a very recent study by Nadeau et al. [107], starting from the computational analysis of the PPI dataset generated in HEK 293 cells by Gordon et al. [17] has revealed processes theoretically affected by the virus [107]. In addition, Verstraete and colleagues [108] also used the SARS-CoV-2 PPI map identified by the Krogan group [17], further expanding this dataset, including other host proteins resulting from structural genomics and interactomics investigations [109,110].

## 5. Perspectives

The studies reviewed here, providing an extensive SARS-CoV-2 network of interactions, allow the research to move in several directions in the fight to contrast the COVID-19 pandemic. In fact, they provide interactors that can be specifically targeted not only by already existing drugs or by drugs in preclinical experimentation with immediate application but also, in a longer range approach, by rationally de novo specifically designed antiviral molecules. Only a deep knowledge of viral life cycle and, in particular, of virus–host PPI can lead to identification of highly specific inhibitors that can be developed in safe and effective antiviral drugs. Complete datasets of three different members of the *Betacoronavirus* genus (SARS-CoV-1, SARS-CoV-2 and MERS-CoV) have been obtained [18] and, as highlighted above (Section 2.1), these findings already identified common viral interactors most likely involved in processes essential for the *Betacoronavirus* genus and therefore potential drug targets also against future emerging pathogens of the *Betacoronavirus* genus. This is of utmost importance in particular in light of the viral high mutation rate already mentioned in the introduction (Section 1). Indeed, the European Center for Disease Prevention and Control (ECDC) recently reported that in the last part of the year 2020, the United Kingdom (UK) has witnessed an unexpected increment in COVID-19 case rates, in particular the London area, in the East and the South-East part of the country [111]. Viral sequence analysis showed that this increase was associated with the emergence, in the above-mentioned areas, of a new variant, named SARS-CoV-2 Variant of Concern, year 2020, month 12, variant 01 (VOC 202012/01), which was previously referred as “Variant under Investigation” (VUI) [111]. The first appearance of the SARS-CoV-2 VOC 202012/01 was identified in a case from 20 September 2020 in the UK [112] and, since then, as of 29 December 2020 a total of 3384 cases of the new variant were reported in the UK, with the SARS-CoV-2 VOC 202012/01 already spreading to Denmark, Portugal, Italy, Iceland, The Netherlands, Spain, Japan, Ireland, India, Israel, Belgium, Australia, Canada, South Korea, Finland, Norway, Hong Kong, Switzerland, Jordan, Germany, France, Sweden, Singapore and Lebanon [111]. The SARS-CoV-2 VOC 202012/01 is characterized by a number of mutations in the structural S protein, in particular deletion HV69-70, deletion Y144, N501Y, A570D, P681H, T716I, S982A, D1118H [113] and by additional mutations in the ORF1ab, ORF8 and N coding regions [114]. Among these mutations, N501Y is localized within the ACE2 receptor binding domain [111] and, in particular, residue 501 is one of six key contact amino acids of the S protein receptor-binding domain [114]. Indeed, a recent study demonstrated that the N501Y mutation increases viral S affinity for the cellular ACE2 receptor [115]. This experimentally determined binding increase might explain the augmented transmissibility that has been observed for the SARS-CoV-2 VOC 202012/01 [111]. Even more concerning are the deletions present in the S coding region of this variant, which might contribute to potential spike surface changes: in particular, deletion HV69-70 has been associated to evasion to the human immune response [114] and deletion Y144 is located in the position where neutralizing antibody 4A8 binds [112]. At the time of writing, there are still no data on the overall effect of the eight listed mutations on the conformation of the S protein of the variant and, therefore, on the potential loss of efficacy against SARS-CoV-2 VOC 202012/01 of the presently administered vaccines which, is worth remembering, all elicit an immune response against the non-mutated SARS-CoV-2 S protein. On the contrary, the strategy focus of this review is to target directly the host proteins in order to prevent interaction with the viral proteins when such interactions are functional for the virus life cycle, as in the case of the of the cellular interactors classified as proviral dependency factor by Gordon and coworkers [18] because their inhibition significantly decreased virus production. In this case, mutations of the viral proteins should have no effect on drug binding on the cellular factors and this strategy should be even more “mutation insensitive” when targeting common viral interactors, most likely involved in processes essential for the *Betacoronavirus* genus, as described at the beginning of this section.

On the same path, it is worth reminding that only three proteins of two different *Alphacoronaviruses* (HCoV-229E and HCoV-NL63) have been used as baits so far [21]. Therefore, it can be finally auspicated that the generation of complete PPI landscapes by *Alphacoronaviruses* might lead to the identification of processes common to all the *Coronaviridae* family, thus identifying common host interactors as valuable druggable targets for the development of pancoronavirus antivirals, effective also against future emerging pathogens of the entire *Coronaviridae* family.

## Figures and Tables

**Table 1:** Proteomics SARS-CoV-2 studies in cellular systems: network and enrichment analysis.

References	Biological Systems	Interactors Identification Methods	PPI Analysis	SARS-CoV-2-Human Interaction Network and Enrichment Analysis (Main Pathways)	Data Availability and Web Resources
Gordon et al. [17]	Transient transfection in HEK-293 cells for PPI studies; Vero E6 cells for drug repurposing.	AP-MS: either N- or C- terminus 2xStrep tagging followed by AP-MS.	SAINTexpress ^(1)^; MiST ^(2)^; Cytoscape; GO ^(3)^ enrichment analysis.	DNA replication, epigenetic and gene-expression regulators, vesicle trafficking, lipid modification, RNA processing and regulation, ubiquitin ligases, signaling, nuclear transport machinery, cytoskeleton, mitochondria and the extracellular matrix.	MS raw data deposited to the PX ^(4)^ Consortium (www.ebi.ac.uk/pride/archive/projects/PXD018117). PPI networks uploaded to NDEx ^(5)^ (https://public.ndexbio.org/#/network/43803262-6d69-11ea-bfdc-0ac135e8bacf).
Gordon et al. [18]	Transient transfection in HEK-293 cells for PPI studies; HeLa cells for IF^5^ experiments; A549-ACE2 and Caco2 cells for validation on viral life cycle; Vero E6 and A549-ACE2 cells for drug repurposing.	AP-MS: either N- or C- terminus 2xStrep tagging followed by AP-MS.	SAINTexpress; MiST; Cytoscape; GO ^(3)^ enrichment analysis.	Regulation of RNA metabolism and ribosome biogenesis, endosomal and Golgi vesicle transport, proteasomal catabolism, cellular response to heat and regulation of intracellular protein transport.	MS-proteomics data deposited to the PX ^(6)^ Consortium (https://www.ebi.ac.uk/pride/archive/projects/PXD021588). PPI networks can be found either in NDEx ^(5)^ and at https://kroganlab.ucsf.edu/network-maps.
Davies et al. [19]	Transient transfection in HEK-293 cells.	AP-MS: either N- (nsp2) or C- (nsp4) terminus FLAG tagging followed by AP-MS.	R statistics software. Cytoscape; GO ^(3)^ enrichment analysis.	Nsp2 interactors are involved in a number of host cell processes, including metabolic processing and transport. Nsp4 interactors showed multiple enriched biological processes, such as cell organization and biogenesis, transport, and metabolic processes.	N/A
Li et al. [20]	Transient transfection in HEK-293 cells. PBMC for proteomic perturbation in COVID-19 patients primary cells.	AP-MS: N- terminus 3xFlag-tagging followed by AP-MS.	MiST; Cytoscape; GO ^(3)^ enrichment analysis.	Inflammation and immune responses, ATP biosynthesis and metabolic processes, nucleotide-excision repair, protein methylation and alkylation, translation initiation, reactive oxygen species metabolic process, ER stress, and mRNA transport.	Datasets deposited to the PX ^(4)^ Consortium (http://proteomecentral.proteomexchange.org) via the iProX partner repository (dataset identifier IPX0002285000).
Stukalov et al. [21]	Lentiviral mediated transduction of A549 cells for PPI identification. A549-ACE2 for both OMICS perturbation and drug repurposing.	AP-MS: C- terminus HA tagging followed by AP-MS.	R statistics software; GO ^(3)^ enrichment analysis.	Stress and DNA damage response, regulation of transcription, cell junction organization, cell survival, motility and innate immune responses.	N/A
Samavarchi-Tehrani et al. [22]	A549 cells transduced by lentiviral constructs (except for nsp1 and nsp3, whose constructs where transfected).	BioID: miniTurbo enzyme fused separately at both N- and C- terminus of each bait, modified proteins purification followed by MS.	SAINTexpress; Cytoscape; GO ^(3)^ enrichment analysis; Humancellmap.org.	Regulation of cell cycle processes, antigen processing, viral genome replication, transcription, regulation of innate immunity, DNA damage checkpoint, histone binding, proteasomal degradation.	Virus–host proximity interactome dataset is available at https://covid19interactome.org/
St-Germain et al. [23]	Stably transfected HEK-293 cells	BioID: BirA* enzyme fused at the N- terminus of 14 viral baits, modified proteins purification followed by MS.	SAINTexpress; Cytoscape; GO ^(3)^ enrichment analysis.	Vesicle-mediated transport, Golgi vesicle transport, ER to Golgi vesicle-mediated transport, response to ER stress, retrograde transport endosome to Golgi, lipid biosynthetic process, ER organization, retrograde vesicle-mediated transport, COPII-coated vesicle budding.	All virus MS data available at https://massive.ucsd.edu
Laurent et al. [24]	Stably transfected HEK-293 cells	BioID: BirA* enzyme fused separately at both N- and C- terminus of each bait, modified proteins purification followed by MS.	ToppCluster; Metascape; GO ^(3)^ enrichment analysis.	Innate immune response, autophagy, apoptosis, lipid metabolism, vesicular transport, chromatin remodeling, mRNA processing, inflammation, viral signal transduction, nucleic acid processing, cell adhesion and migration, platelet activation, coagulation regulation, olfactory receptors homeostasis and olfactory cell signal transmission.	Data exploitation available at http://www.sars-cov-2-interactome.org/

^(1)^ SAINT: Significance Analysis of INTeractome. ^(2)^ MiST: Mass spectrometry interaction STatistics. ^(3)^ GO: Gene Ontology. ^(4)^ PX: ProteomeXchange. ^(5)^ IF immunofluorescence ^(6)^ NDEx: Network Data Exchange.

**Table 3:** Relevant common interactors.

Viral Bait	AP-MS Article	Cellular Preys Identified by AP-MS in at Least Two Different Reports	Preys Found Also by Proximity Labeling			AP-MS/BioID
			Laurent et al. [24]	Samavarchi-Tehrani et al. [22]	St-Germain et al. [23]	
**E**	Gordon et al. [17]	No common interactors	N/A	N/A	N/A	N/A
	Stukalov et al. [21]					
**M**	Gordon et al. [17]	*ATP1B1, COQ8B, INTS4, PITRM1, PMPCB, REEP5, RTN4*	*REEP5, RTN4*	*ATG9A, ATP1B1, REEP5, RTN4*	*PMPCB*	*ATG9AATP1B1, PMPCB, REEP5, RTN4*
	Stukalov et al. [21]	*ARFGEF2, ATG9A, COQ8B, INTS4, PITRM1, PMPCB, RTN4,*				
	Li et al. [20]	*ARFGEF2, ATG9A, ATP1B1, REEP5*				
**N**	Gordon et al. [17]	*G3BP1, G3BP2*	*CAVIN1, G3BP1, G3BP2,*	*CAVIN1, G3BP1, G3BP2,*	Viral bait not tested	*CAVIN1 G3BP1, G3BP2,*
	Stukalov et al. [21]	*CAVIN1, G3BP1, G3BP2*				
	Li et al. [20]	*CAVIN1, G3BP1, G3BP2*				
**S**	Gordon et al. [17]	*GOLGA7, ZDHHC5*	*ZDHHC5*	*ZDHHC5*	No common interactors	*ZDHHC5*
	Stukalov et al. [21]	*GOLGA7, ZDHHC5*				
	Li et al. [20]	No common interactors				
**ORF3a**	Gordon et al. [17]	*ALG5, ARL6IP6, CLCC1, HMOX1, TRIM59, VPS11; VPS-39*	*CLCC1, CPD, HMOX2, VPS39*	*CLCC1, CPD, RAB13, RAB14, TBL2, VPS39*	*CLCC1, VPS-39,*	*CLCC1* *CPD, HMOX2, RAB13, RAB14, TBL2VPS39*
	Stukalov et al. [21]	*CLCC1, CPD, HMOX2, PROCR, RAB13, RAB14, SUMF2, TBL2, VPS11; VPS-39*				
	Li et al. [20]	*ALG5, ARL6IP6, CLCC1, CPD, HMOX1, HMOX2, PROCR, RAB13*, *RAB14, SUMF2, TBL2, TRIM59, VPS-39*				
**ORF6**	Gordon et al. [17]	*RAE1*	*RAE1*	*RAE1*	*RAE1*	*RAE1*
	Stukalov et al. [21]	No interactors identified for this bait				
	Li et al. [20]	*RAE1*				
**ORF7a**	Gordon et al. [17]	*MDN1*	No common interactors	No common interactors	No common interactors	N/A
	Stukalov et al. [21]	*ATR, MDN1*				
	Li et al. [20]	*ATR*				
**ORF8**	Gordon et al. [17]	*GGH*, *NPTX1*, *UGGT*2	*CNNM3*	*CNNM3, GGH, NPTX1, UGGT2,*	*CNNM3*	*GGH, NPTX1, UGGT2,* *CNNM3*
	Stukalov et al. [21]	*CNNM3, GGH, NPTX1, UGGT2*				
	Li et al. [20]	*CNNM3*				
**ORF9b**	Gordon et al. [17]	*TOMM70*	*TOMM70*	*TOMM70*	*TOMM70*	*TOMM70*
	Stukalov et al. [21]	*TOMM70*				
**Nsp1**	Gordon et al. [17]	No common interactors	N/A	N/A	Viral bait not tested	N/A
	Stukalov et al. [21]					
	Li et al. [20]					
**Nsp2**	Gordon et al. [17]	*GIGYF2, RAP1GDS1*	*RAP1GDS1*	*RAP1GDS1,*	No common interactors	*RAP1GDS1*
	Stukalov et al. [21]	*RAP1GDS1*				
	Li et al. [20]	*FOXK1, GIGYF2, RAP1GDS1*				
	Davies et al. [19]	*FOXK1*				
**Nsp3**	Stukalov et al. [21]	No common interactors	N/A	N/A	N/A	N/A
	Li et al. [20]					
**Nsp4**	Gordon et al. [17]	No common interactors	*HSPA5*	No common interactors	*HSPA5*	*HSPA5*
	Stukalov et al. [21]	*HSPA5*				
	Li et al. [20]	No common interactors				
	Davies et al. [19]	*HSPA5*				
**Nsp5**	Gordon et al. [17]	No common interactors	N/A	N/A	Viral bait not tested	N/A
	Li et al. [20]					
**Nsp6**	Gordon et al. [17]	*ATP6AP1, ATP13A3, SIGMAR1*	*ATP6AP1, SIGMAR1,*	*ATP6AP1*	*ATP6AP1*	*ATP6AP1, SIGMAR1*
	Stukalov et al. [21]	*ATP6AP1, ATP13A3, SIGMAR1*				
**Nsp7**	Gordon et al. [17]	No common interactors	N/A	N/A	Viral bait not tested	N/A
	Stukalov et al. [21]					
**Nsp8**	Gordon et al. [17]	*ATE1, HECTD1*	*HECTD1, HERC1*	*HECTD1, HERC1*	Viral bait not tested	*HECTD1* *HERC1*
	Stukalov et al. [21]	*ATE1, HERC1*				
	Li et al. [20]	*HECTD1, HERC1*				
**Nsp9**	Gordon et al. [17]	*EIF4H, GTF2F2, SPART*	*GTF2F2*	No common interactors	Viral bait not tested	*GTF2F2*
	Stukalov et al. [21]	*GTF2F2*				
	Li et al. [20]	*EIF4H, GTF2F2, SPART*				
**Nsp10**	Gordon et al. [17]	No common interactors	N/A	N/A	Viral bait not tested	N/A
	Stukalov et al. [21]					
	Li et al. [20]					
**Nsp12**	Gordon et al. [17]	No common interactors	N/A	N/A	Viral bait not tested	N/A
	Stukalov et al. [21]					
**Nsp13**	Gordon et al. [17]	No common interactors	N/A	N/A	Viral bait not tested	N/A
	Stukalov et al. [21]					
	Li et al. [20]					
**Nsp14**	Gordon et al. [17]	*SIRT5*	*SIRT5*	No common interactors	Viral bait not tested	*SIRT5*
	Stukalov et al. [21]	No common interactors				
	Li et al. [20]	*SIRT5*				
**Nsp15**	Gordon et al. [17]	No common interactors	N/A	N/A	Viral bait not tested	N/A
	Stukalov et al. [21]					
	Li et al. [20]					
**Nsp16**	Gordon et al. [17]	Viral bait not tested	*CCDC22*	No common interactors	Viral bait not tested	*CCDC22*
	Stukalov et al. [21]	No common interactors				
	Li et al. [20]	*CCDC22*				
	Gordon et al. [18]	*CCDC22*				

For each interactor, gene name is reported. This table does not list viral baits studied in only one report that utilized AP-MS for prey detection. In particular, ORF3b, ORF10, ORF9c/ORF14 and Nsp11 were studied only by Gordon and colleagues [17], while data on ORF7b are reported only by Stukalov and colleagues [21]. For each viral protein, the left part of the table lists the reports in which such protein has been used as bait by AP-MS; next, in each line there is the list of cellular preys that were identified interacting by AP-MS with the same bait in at least another report. The central part of the table lists, again for each viral protein, which of the cellular baits (detected by AP-MS in at least two reports) were identified also by proximity labeling in each of the three manuscripts here reviewed. Finally, the right column of the table lists which cellular preys have been found to interact with the respective viral bait in at least two investigations performed by AP-MS and in at least one investigation performed by proximity labeling. In particular, the 12 interactors highlighted in light orange were demonstrated to be either drug targets, or to be part of a complex/process targeted by drug candidates [17] or were host dependency factor [18]. The 11 cellular preys highlighted in grey in the right column of the table have not been tested by the Krogan group because they were not identified as interactors by those authors [17,18].

**Table 4:** Viral bait proteins analyzed by AP-MS and BioID studies.

Viral Proteins		Gordon et al. [17,18]	Stukalov et al. [21]	Li et al. [20]	Davies et al. [19]	Laurent et al. [24]	Samavarchi-Tehrani et al. [22]	St-Germain et al. [23]
1	**M**	✓	✓	✓	-	✓	✓	✓
2	**N**	✓	✓	✓	-	✓	✓	-
3	**E**	✓	✓	-	-	✓	✓	✓
4	**S**	✓	✓	✓	-	✓	✓	✓
5	**ORF3a**	✓	✓ (referred as ORF3)	✓	-	✓	✓	✓
6	**ORF3b**	✓	-	-	-	✓	✓	✓
7	**ORF6**	✓	✓	✓	-	✓	✓	✓
8	**ORF7a**	✓	✓	✓	-	✓	✓	✓
9	**ORF7b**	-	✓	-	-	✓	✓	✓
10	**ORF8**	✓	✓	✓	-	✓	✓	✓
11	**ORF9b**	✓	✓	-	-	✓	✓	✓
12	**ORF10**	✓	-	-	-	✓	-	-
13	**ORF14**	✓(referred as ORF9c)	-	-	-	✓	✓	-
14	**Nsp1**	✓	✓	✓	-	✓	✓	-
15	**Nsp2**	✓	✓	✓	✓	✓	✓	✓
16	**Nsp3**	-	✓	✓	-	✓	✓	✓
17	**Nsp4**	✓	✓	✓	✓	✓	✓	✓
18	**Nsp5**	✓	-	✓	-	✓	✓	-
19	**Nsp6**	✓	✓	-	-	✓	✓	✓
20	**Nsp7**	✓	✓	-	-	✓	✓	-
21	**Nsp8**	✓	✓	✓	-	✓	✓	-
22	**Nsp9**	✓	✓	✓	-	✓	✓	-
23	**Nsp10**	✓	✓	✓	-	✓	✓	-
24	**Nsp11**	✓	-	-	-	-	-	-
25	**Nsp12**	✓	✓	-	-	✓	✓	-
26	**Nsp13**	✓	✓	✓	-	✓	✓	-
27	**Nsp14**	✓	✓	✓	-	✓	✓	-
28	**Nsp15**	✓	✓	✓	-	✓	✓	-
29	**Nsp16**	✓	✓	✓	-	✓	✓	-
	**TOT**	**27**	**24**	**19**	**2**	**28**	**27**	**14**

If a viral protein has been studied in the indicated report, it is marked with the symbol “✓”. If a viral protein has not been studied in the indicated report, it is marked with the symbol “-”. The last line of the table shows the total number of viral proteins studied in each investigation listed in the table.

**Table 5:** FDA approved drugs or compound in clinical trial selected from PPI-based drug repurposing with in vitro and/or in vivo efficacy.

Drug	Chemical Structure	Mechanism of Action	SARS-CoV-2 PPI	Studies Which Detected Virus–Host PPI	In Vitro SARS-CoV-2 Antiviral Effects	In Vivo SARS-CoV-2 Activity
Chlorpromazine FDA approved	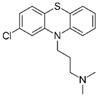	Ligand of Sigma-1 receptor	Nsp6-Sigma-1 ^(1)^	Gordon et al. [17] Stukalov et al. [21] Laurent et al. [24]	A549-ACE2 pIC_50_ = 6.05	Reduced requirement to mechanical ventilation ^(2)^
Fluphenazine FDA approved	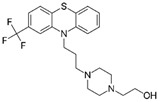	Ligand of Sigma-1 receptor	Nsp6-Sigma-1 ^(1)^	Gordon et al. [17] Stukalov et al. [21] Laurent et al. [24]	A549-ACE2 pIC_50_ = 6.46	Reduced requirement to mechanical ventilation ^(2)^
Haloperidol FDA approved	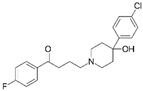	Ligand of Sigma-1 receptor	Nsp6-Sigma-1 ^(1)^	Gordon et al. [17] Stukalov et al. [21] Laurent et al. [24]	A549-ACE2 pIC_50_ = 5.68	Reduced requirement to mechanical ventilation ^(2)^
Indomethacin FDA approved	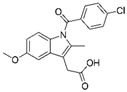	PGES-2 inhibitor	Nsp7-PGES-2 ^(3)^	Gordon et al. [17] Gordon et al. [18]	A549-ACE2 pIC_50_ = 4.25	Reduced hospitalization ^(4)^
Ipatasertib compound in clinical trial	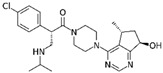	AKT inhibitor a potential kinase phosphorylating SARS-CoV-2 protein N	N-M ^(5)^ and M-AKT ^(6)^ kinase	^(5)^ Li et al. [20] ^(6)^ Laurent et al. [24]	A549-ACE2 5 μM	N/A

^(1)^ Interaction detected also in the SARS-CoV-1 interactome [18,21] ^(2)^ Retrospective real-world clinical data analysis; a reduced requirement to mechanical ventilation was observed in COVID-19 patients taking antipsychotics drugs targeting Sigma1 in comparison to COVID-19 patients taking antipsychotics drugs not targeting Sigma1 [18]. ^(3)^ Conserved interaction detected also in the SARS-CoV-1 and in the MERS-CoV interactome [18]. ^(4)^ Non-interventional study; reduced hospitalization of COVID-19 patients users of indomethacin targeting PGES-2 in comparison to COVID-19 patients users of anti-inflammatory drug Celecoxib not targeting PGES-2 which does not have anti–SARS-CoV-2 activity in vitro [18]. ^(5)^ An N-M intraviral PPI interaction was detected by Li et al. [20] and ^(6)^ an M-AKT2 interaction was detected by Laurent et al. [24].
